# *Bacteroides uniformis* combined with fiber amplifies metabolic and immune benefits in obese mice

**DOI:** 10.1080/19490976.2020.1865706

**Published:** 2021-01-26

**Authors:** Inmaculada López-Almela, Marina Romaní-Pérez, Clara Bullich-Vilarrubias, Alfonso Benítez-Páez, Eva M. Gómez Del Pulgar, Rubén Francés, Gerhard Liebisch, Yolanda Sanz

**Affiliations:** a Microbial Ecology, Nutrition & Health Research Unit. Institute of Agrochemistry and Food Technology, Spanish National Research Council (IATA-CSIC), Valencia, Spain; b CIBERehd, Hospital General Universitario, Alicante, Spain; Dpto. Medicina Clínica, Universidad Miguel Hernández, San Juan, Spain; c Institute of Clinical Chemistry and Laboratory Medicine, University Hospital Regensburg, Regensburg, Germany

**Keywords:** Obesity, dietary fiber, microbiota, intraepithelial lymphocytes, innate lymphoid cells

## Abstract

Gut microbiota represents a therapeutic target for obesity. We hypothesize that *B. uniformis* CECT 7771 combined with wheat bran extract (WBE), its preferred carbon source, may exert superior anti-obesity effects. We performed a 17-week intervention in diet-induced obese mice receiving either *B. uniformis*, WBE, or their combination to identify interactions and independent actions on metabolism and immunity. *B. uniformis* combined with WBE was the most effective intervention, curbing weight gain and adiposity, while exerting more modest effects separately. The combination restored insulin-dependent metabolic routes in fat and liver, although the bacterium was the primary driver for improving whole-body glucose disposal. Moreover, *B. uniformis*-combined with WBE caused the highest increases in butyrate and restored the proportion of induced intraepithelial lymphocytes and type-3 innate lymphoid cells in the intestinal epithelium. Thus, strengthening the first line of immune defense against unhealthy diets and associated dysbiosis in the intestine. This intervention also attenuated the altered IL22 signaling and liver inflammation. Our study shows opportunities for employing *B. uniformis*, combined with WBE, to aid in the treatment of obesity.

## Introduction

Obesity is a leading public health problem given its high prevalence and associated complications [e.g., type 2 diabetes (T2D) and cardiovascular diseases]. A deeper understanding of the multiple factors controlling body weight is urgently needed to tackle obesity. Gut microbiota has been identified as a key factor that, through interactions with the diet and intestinal immunity, influence metabolic health.^1–3^ Western diets (low-fiber, high-fat and simple sugars) induce gut-microbiota imbalances (dysbiosis)^4,5^ and damage intestinal barrier integrity and immune homeostasis.^6,7^ Alterations to intestinal microbiota and leakage of their components (cells, proteins, and metabolites) across the intestinal barrier, permeabilized by unhealthy diets, contributes to obesity-associated low-grade inflammation and insulin resistance in metabolic tissues.^7,8^

Moreover, investigations revealing interactions between diet, gut microbiota, and host metabolism^9,10^ have allowed the identification of key intestinal bacteria that represent new targets to improve metabolic health.^11^

For instance, *Bacteroides uniformis* CECT 7771 reduced the high-fat diet-induced metabolic alterations while improving antigen-presentation by dendritic cells, impacting on CD4 + T-cell proliferation in an obesity model.^12^ On the other hand, fiber serves as fuel for commensal intestinal bacteria, promoting their growth and metabolic activity, which could also mediate some of the fiber-induced benefits on human metabolism.^13,14^ Indeed, high intake of dietary fiber is known to help in weight maintenance and reduce the risk of coronary heart disease and T2D.^15^ Furthermore, *Bacteroides* spp. thrive in fiber-enriched environments as they have complex enzymatic machinery to utilize oligo- and poly-saccharides as nutrients.^16-17^ *B. uniformis* CECT 7771 preferred carbon source was evaluated *in vitro*, thus maximizing potential benefits of their combined administration. This could also help lower the effective bacterial dose required, overcoming the challenge of producing intestinal anaerobes at high cell densities. Compared to other carbon sources, *B. uniformis* CECT 7771 thrived on wheat-bran extract (WBE), enriched in arabinoxylan oligosaccharides (AXOS).^16^ Intervention studies in humans reveal that wheat-derived AXOS have multiple metabolic benefits,^13^ especially on the maintenance of glucose homeostasis^17^ and also exert a bifidogenic effect and promote the abundance of butyrate-producing bacteria.^18^

Here, we hypothesized that *B. uniformis* CECT 7771 combined with WBE could have an additive impact on restoring the metabolic and immune alterations of diet-induced obesity and exert greater effects than either WBE or the bacterium alone in mice. Specifically, we assessed whether combined intervention protects against the Western diet-induced intestinal immune impairment, which is one of the causative mechanisms underlying metabolic dysfunction in obesity.

## Results

### *B. uniformis* and WBE amplified the reduction of weight-gain in mice fed HFHSD

**Figure 1.**
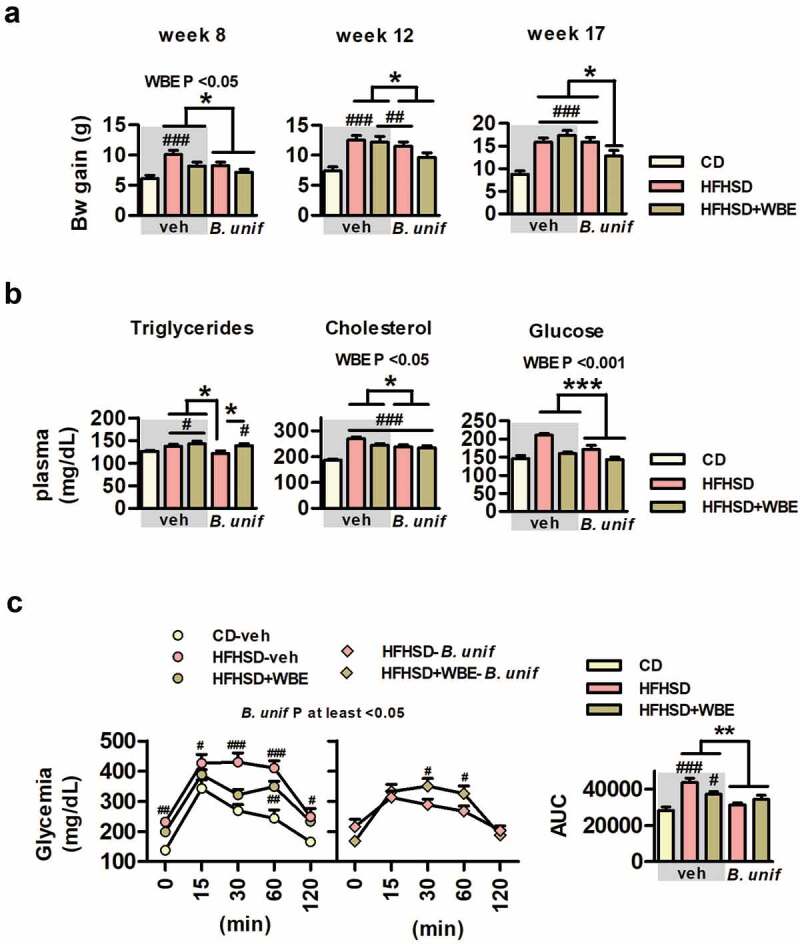
*B. uniformis* CECT 7771 combined with WBE reduces weight gain in mice fed HFHSD. Bw gain, triglycerides and OGTT of controls (CD fed mice receiving vehicle, CD-veh) and HFHSD-fed mice receiving vehicle, WBE, *B. unif* or the combination of both: HFHSD-veh, HFHSD+WBE, HFHSD-*B. unif* or HFHSD+WBE-*B. unif*. a. Bw gain at week 8 (two-way ANOVA in HFHSD-fed groups: *B. unif* effect or WBE effect P <0.05), at week 12 (two-way ANOVA in HFHSD-fed groups: *B. unif* effect P <0.05) and at week 17 (two-way ANOVA in HFHSD-fed groups: *B. unif* x WBE interaction P <0.05; *post hoc* P <0.05). N = 10 for all groups b. Triglycerides (Kruskal-Wallis: HFHSD-*B. unif* vs HFHSD-veh, HFHSD+WBE or HFHSD+WBE-*B. unif* P <0.05), cholesterol and glucose concentrations in plasma (two-way ANOVA in HFHSD-fed groups: WBE and *B. unif* effect P <0.05 and P <0.001, respectively) after 12 weeks of HFHSD feeding. Triglycerides: CD-veh N = 8; HFHSD-veh N = 9; HFHSD+WBE and HFHSD+WBE-*B. unif* N = 10 and HFHSD-*B. unif* N = 7; cholesterol: CD-veh and HFHSD-veh N = 8; HFHSD+WBE and HFHSD+WBE-*B. unif* N = 10 and HFHSD-*B. unif* N = 7. Glucose: CD-veh and HFHSD-veh N = 9, HFHSD+WBE, HFHSD-*B. unif* and HFHSD+WBE-*B. unif* = 10 c. OGTT (two-way ANOVA in HFHSD-fed groups: *B. unif* effect P at least <0.05 vs HFHSD-fed mice that did not receive the bacteria) and AUC (two-way ANOVA in HFHSD-fed groups: *B. unif* effect P <0.01) at week 14. CD-veh, HFHSD-veh, HFHSD-*B. unif* and HFHSD+WBE-*B. unif* N = 10 and HFHSD+WBE N = 8 Data were represented as the mean ± SEM. Statistical differences between HFHSD-fed groups (receiving or not the bacterium and/or WBE) and controls (CD-veh) were analyzed using one-way ANOVA followed by post-hoc Tuckey. ^#^P <0.05, ^##^P <0.01, ^###^P <0.001 vs control group. *P <0.05, **P <0.01 and ***P <0.001 indicate differences within HFHSD-fed groups. AUC, area under the curve; *B. unif, Bacteroides uniformis* CECT 7771; Bw, body weight; CD, control diet; HFHSD, high-fat high-sugar diet; WBE, wheat-bran extract

At week 8 body-weight gain induced by high-fat high-sugar diet (HFHSD) was lower when *B. uniformis* and/or WBE were administered (Figure 1a). Nonetheless, these effects diminished over time, especially in mice fed WBE alone, while the downward trend continued in those administered *B. uniformis* alone or combined with WBE until week 12, with *B. uniformis* and WBE inducing a greater impact on the prevention of weight gain comparatively until week 17 (Figure 1a and Figure S2A). Compared to controls, HFHSD-fed mice showed a slight increase in fasting triglycerides in plasma, cholesterol and glucose (Figure 1b) and impaired OGTT (Figure 1c). WBE reduced cholesterol and normalized glycemia but did not affect triglycerides (Figure 1b). By contrast, *B. uniformis* alone significantly reduced triglycerides in HFHSD-fed mice. Alone or combined with WBE, this bacterium also diminished plasma cholesterol and glucose (Figure 1b) and ameliorated oral glucose intolerance of HFHSD-fed mice and the area under the curve (AUC) (Figure 1c). By contrast, compared to controls, AUC remained elevated in HFHSD-fed mice receiving WBE alone. This suggests that *B. uniformis* rather than WBE improved whole glucose clearance in the OGTT. Insulin plasma levels remained unaffected whatever the treatment (Figure S2B). Neither glucagon-like peptide-1 (GLP-1) nor peptide YY (PYY) in plasma was significantly affected by HFHSD; however, the bacterium–WBE combination normalized GLP-1 levels compared to untreated HFHSD-fed mice and those receiving *B. uniformis* or WBE separately, while *B. uniformis* increased PYY in mice compared to controls (Figure S2B).

### *B. uniformis* CECT 7771 combined with WBE reduces epididymal fat and restores insulin-dependent metabolic routes in adipose tissue and liver in mice fed HFHSD

**Figure 2.**
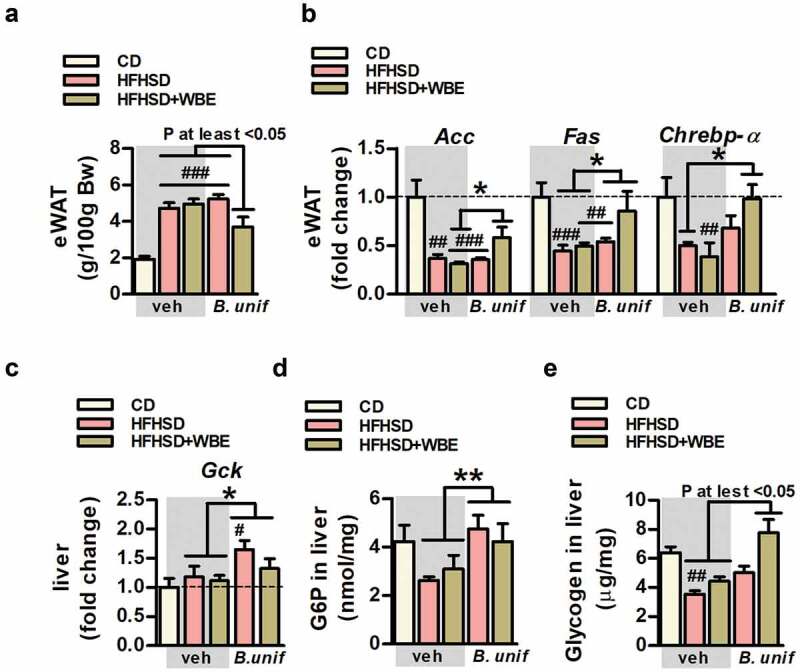
*B. uniformis* CECT 7771 combined with WBE curbs fat gain and normalizes insulin-dependent metabolic routes in adipose tissue and liver in HFHSD-fed mice. Weight of epididymal WAT, levels of expression of *de novo* lipogenesis-related genes in epididymal WAT, correlation between these genes and adiposity and glucose metabolism-related markers in liver of controls (CD-fed mice receiving vehicle, CD-veh) and HFHSD-fed mice receiving vehicle, WBE, *B. unif* or the combination of both: HFHSD-veh, HFHSD+WBE, HFHSD-*B. unif* or HFHSD+WBE-*B. unif* a. Weight of epididymal WAT at week 17 (two-way ANOVA in HFHSD-fed groups: *B. unif* x WBE interaction P <0.01, *post hoc* P at least <0.05). N = 10 for all groups b. Gene expression of *Acc* (two-way ANOVA in HFHSD-fed groups: *B. unif* x WBE interaction P <0.05; *post hoc* P <0.05 compared to HFHSD+WBE), *Fas* (two-way ANOVA in HFHSD-fed groups: *B. unif* effect P <0.05) and *Chrebpα* (two-way ANOVA in HFHSD-fed groups: *B. unif* x WBE P <0.05; *post hoc* P <0.05 compared to HFHSD-veh) in epididymal WAT at week 17. *Acc*: CD-veh N = 6; HFHSD-veh, HFHSD+WBE and HFHSD+WBE-*B. unif* N = 7 and HFHSD-*B. unif* N = 8. *Fas*: CD-veh N = 6; HFHSD-veh, HFHSD+WBE and HFHSD-*B. unif* N = 8 and HFHSD+WBE-*B. unif* N = 7. *Chrebpα*: CD-veh and HFHSD-veh N = 5; HFHSD+WBE and HFHSD+WBE-*B. unif* N = 7 and HFHSD-*B. unif* N = 6 c. Gene expression of *Gck* at week 17 in liver (two-way ANOVA in HFHSD-fed groups: *B. unif* effect P <0.05). *Gck*: CD-veh, HFHSD-veh, HFHSD-*B. unif* and HFHSD+WBE-*B. unif* N = 8 and HFHSD+WBE N = 6 d. G6P concentration in liver at week 17 (two-way ANOVA in HFHSD-fed mice; *B. unif* effect P <0.01). CD-veh and HFHSD-veh N = 9, HFHSD+WBE N = 7 and HFHSD-*B. unif* and HFHSD+WBE-*B. unif* N = 9 e. Glycogen concentration in liver at week 17 (Kruskal-Wallis test P at least <0.05 vs HFHSD-veh). CD-veh and HFHSD-veh N = 9; HFHSD+WBE N = 7 and HFHSD-*B. unif* and HFHSD+WBE-*B. unif* N = 8 Data were represented as the mean ± SEM. Statistical differences between HFHSD-fed groups (receiving or not the bacterium and/or WBE) and controls (CD-veh) were analyzed using one-way ANOVA followed by post-hoc Tuckey. ^#^P <0.05, ^##^P <0.01, ^###^P <0.001 vs control group. *P <0.05 and **P <0.01 indicate differences within HFHSD-fed groups. *Acc*, acetyl-CoA carboxylase; *B. unif, Bacteroides uniformis* CECT 7771; Bw, body weight; *Chrebpα*, Carbohydrate-responsive element-binding protein-alpha; CD, control diet; eWAT, epididymal white adipose tissue; *Fas*, fatty acid synthase G6P, glucose-6-phosphate; *Gck*, glucokinase; *Glut2*, glucose transporter 2; HFHSD, high-fat high-sugar diet; *Hsl*, hormone sensitive lipase; *Lpl*, lipoprotein lipase; veh, vehicle; WBE, wheat bran extract

Compared to controls, epididymal fat was higher in untreated mice fed HFHSD and those receiving *B. uniformis* or WBE separately (Figure 2a). This increased visceral adiposity was only attenuated by the bacterium–WBE combination (Figure 2a). The expression of lipoprotein lipase (*Lpl*) or the hormone-sensitive lipase (*Hsl*) remained unaffected in epididymal WAT of mice fed HFHSD whatever the treatment (Figure S3A) but the lipogenic genes, acetyl-CoA carboxylase (*Acc*) and fatty acid synthase (*Fas*) were reduced after HFHSD feeding (Figure 2b). Separately, neither the bacteria nor WBE modified *Acc* in obese mice, while only the combination of both attenuated HFHSD-induced lipogenesis inhibition, as reflected in the normalization of *Acc* mRNA expression (Figure 2b). Overall, *B. uniformis* raised *Fas* expression in epididymal WAT compared to untreated obese mice but this effect was strengthened by its combination with WBE (Figure 2b). The expression of glucose transporter 4 (*Glut4*) in epididymal WAT was unaltered in HFHSD-fed mice whatever the treatment (Figure S3B). Nevertheless, compared to controls, the carbohydrate responsive-element-binding protein α (*Chrebpα*), a transcriptional factor stimulating *Acc* and *Fas* to induce *de novo* lipogenesis using glucose-derived metabolites as precursors, were reduced by HFHSD-feeding, reaching statistical significance in WBE-treated mice fed HFHSD (Figure 2b). The bacteria and the fiber interacted, normalizing *Chrebpα* expression compared to untreated mice fed HFHSD.

In liver, the expression of the glucose transporter 2 (*Glut2*) (Figure S3C) and glucokinase (*Gck*) (Figure 2c) were unaltered by HFHSD. However, *B. uniformis*, with or without WBE, increased *Gck* expression (Figure 2c) as well as glucose-6-phosphate (G6P) levels (Figure 2d), a product of *Gck* that stimulates glycogenogenesis, compared to HFHSD-fed groups not administered the bacteria. Liver glycogen was reduced in untreated HFHSD-fed mice (Figure 2e) and strongly increased by the *B. uniformis*-WBE combination, reaching levels close to those of controls. The increased G6P in liver, which is also a precursor for lipogenesis, did not affect this route as suggested by unchanged hepatic levels of lipogenic genes (*Acc* and *Fas*, Figure S3D), triglycerides (Figure S3E) and free fatty acids (Figure S3F).

### Visceral fat thermogenesis normalized by bacteria-WBE combination in mice fed HFHSD

**Figure 3.**
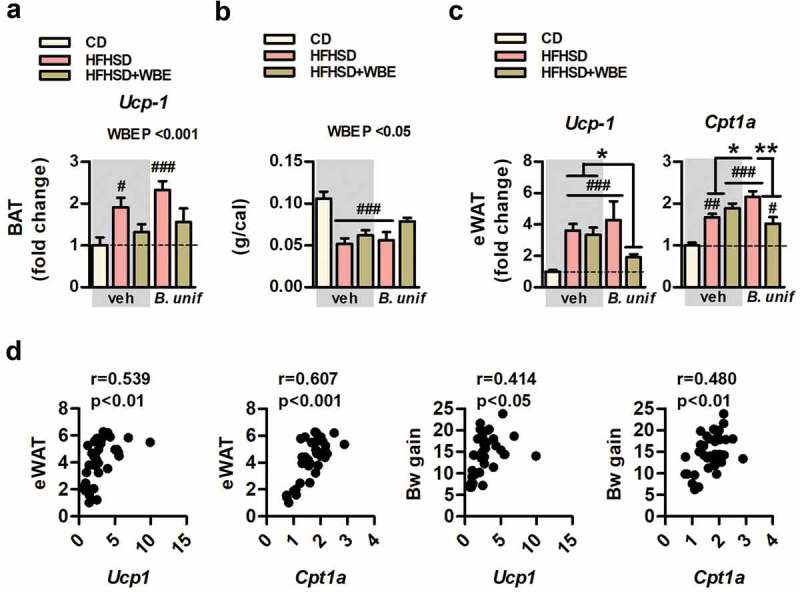
*B. uniformis* CECT 7771 combined with WBE restores the obesity-associated increased expression of *Ucp-1* in epididymal fat which correlates with a healthier metabolic phenotype. *Ucp-1 gene* expression in BAT, food efficiency, *ucp-1* and c*pt1a* gene expression in epididymal WAT, correlation between *ucp-1* and *cpt1a* in epididymal WAT to adiposity or to Bw gain of controls (CD-fed mice receiving vehicle, CD-veh) and HFHSD-fed mice receiving vehicle, WBE, *B. unif* or the combination of both: HFHSD-veh, HFHSD+WBE, HFHSD-*B. unif* or HFHSD+WBE-*B. unif a. Ucp-1 gene* expression pattern in BAT (two-way ANOVA in HFHSD-fed groups: WBE effect *P* < .001) at week 17. N = 10 for all groups b. Food efficiency at week 6 (two-way ANOVA in HFHSD-fed groups: WBE effect *P* < .05). N = 10 for all groups c. *Ucp-1* (Kruskal-Wallis test HFHSD+WBE-*B. unif* vs HFHSD+WBE and HFHSD-veh *P* < .001) and *Cpt1-a* (two-way ANOVA in HFHSD-fed groups: *B. unif* x WBE interaction *P* < .01, *post hoc* P at least <0.05) gene expression of in epididymal WAT at week 17. *Ucp-1*: CD-veh N = 6; HFHSD-veh, HFHSD+WBE and HFHSD+WBE-*B. unif* N = 8 and HFHSD-*B. unif* N = 7. *Cpt1-a*: CD-veh and HFHSD+WBE-*B. unif* N = 7 and HFHSD-veh, HFHSD+WBE and HFHSD-*B. unif* N = 8 d. Bravais-Pearson´s correlation between *Ucp-1* or *Cpt1-a* expression in epididymal WAT to epididymal WAT weight or to Bw gain, N = 38 Data were represented as the mean ± SEM. Statistical differences between HFHSD-fed groups (receiving or not the bacterium and/or WBE) and controls (CD-veh) were analyzed using one-way ANOVA followed by post-hoc Tuckey ^#^P < .05, ^##^P < .01, ^###^P < .001 vs control group. *P < .05 and **P < .01 indicate differences within HFHSD-fed groups. *B. unif, Bacteroides uniformis* CECT 7771; BAT, brown adipose tissue; Bw, body weight; CD, control diet; *Cpt1-a*, carnitine palmitoyltransferase 1a; eWAT, epididymal white adipose tissue; HFHSD, high-fat high-sugar diet; *Ucp-1*, uncoupled protein-1; veh, vehicle; WBE, wheat bran extract

Thermogenesis in the brown adipose tissue (BAT) was enhanced in HFHSD-fed mice as suggested by the increased expression of the uncoupling protein-1 (*Ucp-1*) after HFHSD-feeding (Figure 3a). WBE, but not *B.uniformis*, normalized the expression of Ucp-1 in HFHSD-fed mice. Food efficiency, an indirect measure of energy expenditure, was reduced by week-6 of HFHSD-feeding (Figure 3b) which was normalized by WBE, combined or not with *B. uniformis*. Although *Ucp-1* abundance was much lower in epididymal fat compared to BAT, its expression also showed a 3-fold increase in untreated mice fed HFHSD (Figure 3c). While *Ucp-1* remained high in mice fed HFHSD receiving either *B. uniformis* or WBE, their combination interacts to normalize *Ucp-1* (Figure 3c). The mRNA levels of carnitine palmitoyltransferase 1a (*Cpt1-a*), the rate-limiting enzyme of fatty acid oxidation and normally coupled with thermogenesis in the adipose tissue, was also increased in untreated mice fed HFHSD and remained high in either *B. uniformis* or WBE-treated mice and, to a lesser extent, in those receiving the combination of both (Figure 3c). Positive correlations between *Ucp-1* or *Cpt1-a* to epididymal WAT or weight gain were identified (Figure 3d).

### B. uniformis CECT 7771 and WBE administration modifies gut microbiota, while cecal abundance of fatty acids is mainly affected by B. uniformis CECT 7771 in mice fed HFHSD

Alpha diversity descriptor analysis did not show a major effect of HFHSD on the cecal microbiota structure compared to controls. However, the WBE reduced diversity while *B. uniformis* increased diversity, and their combination restored diversity metrics to control levels (Figure S4A). The multivariate dbRDA analysis showed how treatments affected the microbial community structure at OTUs level. The dbRDA1 dimension better discriminated WBE-treated groups, whereas dbRDA2 showed the HFHSD shifted microbiota structure and this change was partially restored by *B. uniformis* administration (Figure S4B). The interventions also influenced OTUs abundance (potential bacterial species) identified among groups (*p* < .001). Specifically, the HFHSD caused a depletion of *Alistipes* spp. (OTU117), *Odoribacter* spp. (OTU124), *Roseburia* spp. (OTU87) and *Christensenella* spp. (OTU114), which was restored efficiently only by *B. uniformis* (Figure S4C). WBE increased *Bifidobacterium* species abundance (OTU102) and, particularly, Lachnospiraceae species including those belonging to Lachnospiraceae NK4A136 and the genus *Blautia* (OTU273). Concomitantly, WBE reduced the proportion of certain Ruminococcaceae and Oscillospiraceae species together with *Bilophila* spp (OTU22) (Figure S4C). WBE and *B. uniformis* combined administration increased several Lachnospiraceae (OTU30, OTU180, OTU415, OTU76, OTU59, OTU155) and Muribaculaceae (OTU214) species, compared to the other groups (corrected *p* < .05).

**Figure 4.**
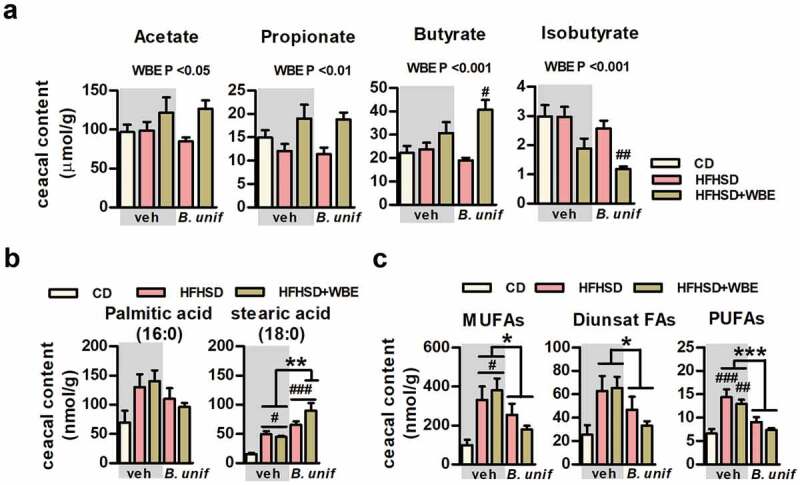
*B. uniformis* CECT 7771, combined or not with WBE, differently influences cecal concentrations short- and long-chain fatty acids. Levels of SCFAs, saturated and unsaturated long-chain FAs in the cecal content of controls (CD-fed mice receiving vehicle, CD-veh) and HFHSD-fed mice receiving vehicle, WBE, *B. unif* or the combination of both: HFHSD-veh, HFHSD+WBE, HFHSD-*B. unif* or HFHSD+WBE-*B. unif* A. Concentrations of short-chain fatty acids (SCFAs, acetate, propionate, butyrate and isobutyrate) in the cecum (nmol/g of dry weight of cecal content) at week 17 (two-way ANOVA in HFHSD-fed groups: WBE effect P at least <0.05). CD-veh N = 8 and HFHSD-veh, HFHSD+WBE, HFHSD-*B. unif* and HFHSD+WBE-*B. unif* N = 10 B. Cecal levels of saturated fatty acids (palmitic and stearic acids, 16:0 and 18:0, respectively; nmol/g of dry weight of cecal content) (for stearic acid, two-way ANOVA in HFHSD-fed groups: *B. unif* x WBE interaction *P* < .05; *post hoc P* < .01 vs HFHSD-veh or HFHSD+WBE) at week 17. FA 16:0: CD-veh N = 8 and HFHSD-veh, HFHSD+WBE, HFHSD-*B. unif* and HFHSD+WBE-*B. unif* N = 10. FA 18:0 CD-veh N = 8; HFHSD-veh and HFHSD-*B. unif* N = 10; HFHSD+WBE N = 7 and HFHSD+WBE-*B. unif* N = 9 C. Concentrations of unsaturated fatty acids (total MUFAs, diunsaturated FAs and PUFAs) in cecum (nmol/g of dry weight of cecal content) at week 17 (two-way ANOVA in HFHSD-fed groups: *B. unif* effect P at least <0.05) MUFAs: CD-veh N = 8; HFHSD-veh, HFHSD-*B. unif* and HFHSD+WBE-*B. unif* N = 10 and HFHSD+WBE N = 9. Diunsaturated FAs and PUFAs: CD-veh N = 7 and HFHSD-veh, HFHSD+WBE, HFHSD-*B. unif* and HFHSD+WBE-*B. unif* N = 10 Data were represented as the mean ± SEM. Statistical differences between HFHSD-fed groups (receiving or not the bacterium and/or WBE) and controls (CD-veh) were analyzed using one-way ANOVA followed by post-hoc Tuckey ^#^P < .05, ^##^P < .01, ^###^P < .001 vs control group. *P < .05, **P < .01 and ***P < .001 indicate differences within HFHSD-fed groups. *B. unif, Bacteroides uniformis* CECT 7771; CD, control diet; Diunsat FAs, diunsaturated fatty acids; HFHSD, high-fat high-sugar diet; MUFAs, monounsaturated fatty acids; PUFAs, polyunsaturated fatty acids; veh, vehicle and WBE, wheat bran extract

In accordance with WBE increasing SCFAs-producing species (Lachnospiraceae NK4A136 group and *Blautia* genus), the actual concentration of acetate, propionate, and butyrate were increased while that of iso-butyrate was reduced in the cecal content of mice fed HFHSD receiving the WBE (Figure 4a). Nevertheless, the butyrate increase was higher in HFHSD-fed mice receiving the combination of WBE and *B. unformis*, suggesting an additive effect in the production of this key metabolite (Figure 4a).

Cecal concentrations of either palmitic (16:0) or stearic (18:0) acid, the most abundant dietary saturated fatty acids (FAs) (Table S1), were unchanged or increased, respectively, in HFHSD-fed mice untreated or treated (with *B. uniformis* or WBE) compared to controls (Figure 4b). Under HFHSD, *B. uniformis* and WBE, in combination, interacted increasing stearic (18:0) (Figure 4b) and arachidic acid (20:0) (Figure S5A). Similarly, concentrations of total monounsaturated FAs (MUFAs) [mainly 10-heptadecenoic acid (17:1, n-7), oleic acid (18:1, n-9) and vaccenic acid (17:1, n-7), Figure S5B] and polyunsaturated FAs (PUFAs) [mostly dihomo-ɤ-linolenic (20:3, n-6), arachidonic (20:4, n-6), eicosapentaenoic (20:5, n-3), adrenic (22:4, n-6), docosapentaenoic (22:5, n-3), and docosahexaenoic (22:6, n-3) acid, Figure S5D] were higher in the cecal content after HFHSD-feeding compared to controls (Figure 4c). Independently of WBE, *B. uniformis* reduced cecal MUFAs (Figure 4c and online Figure S5B), diunsaturated FAs (Figure 4c and Figure S5C) and PUFAs (Figure 4c and Figure S5D) compared to mice not administered the bacteria (untreated and WBE-treated mice fed HFHSD).

### *B. uniformis CECT 7771* combined with WBE prevents the HFHSD-associated pro-inflammatory shift in the gut

To unravel mechanisms through which *B. uniformis* combined with WBE improved the metabolic phenotype of HFHSD-fed mice, we examined intestinal barrier integrity-related mediators. In colon, the expression of antimicrobial peptides, i.e. lysozyme 1 (*Lyz1*), regenerating islet-derived protein 3 gamma (*Reg3ɤ*), and phospholipase A2 group IIA (*Pla2g2a*) or the proliferator marker *Ki67* remained unchanged regardless of treatment (Figure S6A). In ileum, the analysis of tight junction protein expression showed reduced *occludin* in HFHSD-fed mice (Figure S6B). Separately, neither *B. uniformis* nor WBE reversed this reduction but their combination increased *occludin* expression in HFHSD-fed mice. *Occludin* in ileum negatively correlated to weight gain and adiposity and positively to hepatic glycogen (Figure S6C). As we demonstrated that the combination of *B. uniformis* and WBE has an additive effect preventing body weight gain and visceral adiposity, we further analyzed key intestinal immune cell populations in this intervention group and compared it to controls and untreated mice fed HFHSD.

**Figure 5.**
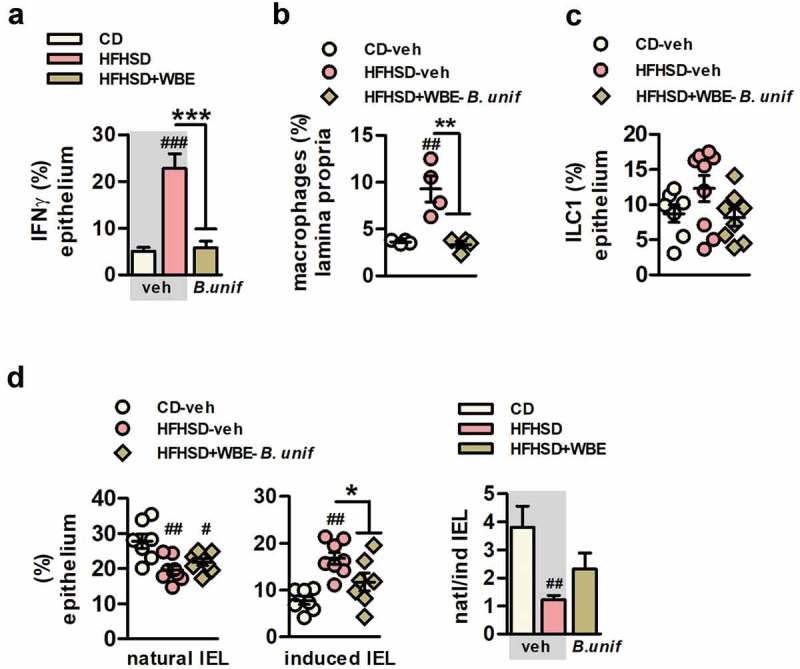
*B. uniformis* CECT 7771 combined with WBE protects against the intestinal immune imbalance of HFHSD-fed mice, curbing induced IEL and increasing ILC3 in the intestinal epithelium. A. IFNɤ+ intestinal epithelial cells of the total intestinal epithelial cells (percentage) at week 17 in controls (CD-fed mice receiving vehicle, CD-veh) and HFHSD-fed mice receiving vehicle or the combination of *B. uniformis* and WBE: HFHSD-veh and HFHSD+WBE-*B. unif*. N = 8 for all groups B. Macrophages in lamina propria (percentage) at week 17 in controls (CD-fed mice receiving vehicle, CD-veh N = 7) and HFHSD-fed mice receiving vehicle or the combination of *B. uniformis* and WBE. N = 4 C. ILC1 of the total intestinal epithelial cells (percentage) week 17 in controls (CD-fed mice receiving vehicle, CD-veh) and HFHSD-fed mice receiving vehicle or the combination of *B. uniformis* and WBE: HFHSD-veh and HFHSD+WBE-*B. unif*. ILC1: CD-veh N = 7; HFHSD-veh N = 9 and HFHSD+WBE N = 8. D. Natural and induced IEL (percentage of total intestinal epithelial cells and ratio) in the intestinal epithelium at week 17 in controls (CD-fed mice receiving vehicle, CD-veh N = 7) and HFHSD-fed mice receiving vehicle or the combination of *B. uniformis* and WBE: HFHSD-veh N = 8 and HFHSD+WBE-*B. unif* N = 8 Data were represented as the mean ± SEM. one-way ANOVA followed by *post hoc* Tuckey was conducted for panels C-E. ^#^P < .05, ^##^P < .01, ^###^P < .001 vs control group. *P < .05, **P < .01 and ***P < .001 indicate differences within HFHSD-fed groups (fed or not WBE). *B. unif, Bacteroides uniformis*; Bw, body weight; CD, control diet; epididymal WAT, epididymal white adipose tissue; HFHSD, high-fat high-sugar diet; IEL, intraepithelial lymphocytes; ILC, innate lymphoid cells; WBE, wheat bran extract

Untreated obese mice showed increased intracellular levels of IFNɤ in the epithelium of small intestine (Figure 5a) indicating that HFHSD-feeding induced a pro-inflammatory shift in the gut. The analysis of INFɤ-producing cells revealed that, compared to controls, HFHSD also led to a higher abundance of macrophages in lamina propria, with similar levels of type 1 and type 2 macrophages (Figure S6D). In the intestinal epithelium, HFHSD-feeding did not impact on innate lymphoid cells (ILC) type 1 (Figure 5c). Nevertheless, intraepithelial lymphocytes (IEL) were altered, showing reduced levels of natural IEL but higher induced IEL (Figure 5d) which highlights a primary role of induced IEL on IFNɤ production as a consequence of HFHSD-feeding. *B.uniformis* combined with WBE seemed to alleviate the intestinal proinflammatory state since this intervention restored the levels of IFNɤ in the epithelium (Figure 5a) and normalized macrophages in the lamina propria (Figure 5b) and the levels of induced IEL in the epithelium of the small intestine (Figure 5d)

### *B. uniformis* CECT 7771 combined with WBE ameliorates HFHSD-associated systemic inflammation through IL22 signaling

**Figure 6.**
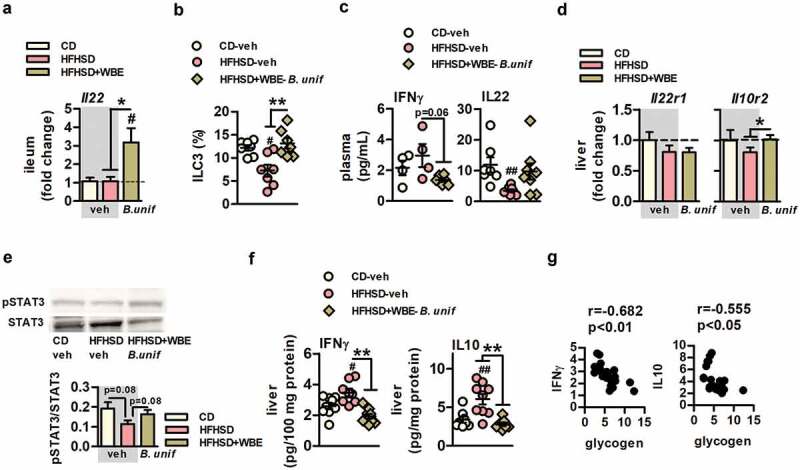
*B. uniformis* CECT 7771 combined with WBE ameliorates obesity-associated systemic inflammation in HFHSD-fed mice. Plasma and hepatic levels of inflammatory markers of controls (CD-fed mice receiving vehicle, CD-veh) and HFHSD-fed mice receiving vehicle or the combination of *B. uniformis* and WBE: HFHSD-veh and HFHSD+WBE-*B. unif A. Il22* gene expression in ileum. CD-veh and HFHSD-veh N = 6 and HFHSD+WBE-*B. unif* N = 7 B. ILC3 of the total intestinal epithelial cells (percentage) week 17 in controls (CD-fed mice receiving vehicle, CD-veh) and HFHSD-fed mice receiving vehicle or the combination of *B. uniformis* and WBE. N = 7 C. IFNɤ and IL22 plasma concentrations (pg/mL) at week 17. IFNɤ: CD-veh and HFHSD-veh N = 4 and HFHSD+WBE-*B. unif* N = 6. IL22: CD-veh N = 7; HFHSD-veh N = 9 and HFHSD+WBE-*B. unif* N = 8 *D. Il22r1* and *Il10r* gene expression in liver. CD-veh N = 6 and HFHSD-veh and HFHSD+WBE-*B. unif* N = 8 E. IL22 intracellular signaling (pSTAT3/STAT3 ratio) at week 17. CD-veh and HFHSD-veh N = 8 and HFHSD+WBE-*B. unif* N = 9 F. IFNɤ and IL10 concentrations (pg/100 mg protein) in liver at week 17. IFNɤ: CD-veh N = 8; HFHSD-veh N = 9 and HFHSD+WBE-*B. unif* N = 7. IL10 CD-veh and HFHSD+WBE-*B. unif* N = 9 and HFHSD-veh N = 8 G. Bravais-Pearson´s correlation between between IL10 or IFNɤ and glycogen hepatic depots. N = 22 Data were represented as the mean ± SEM. Panels A, C and D were assessed by one-way ANOVA followed by *post hoc* Tuckey, while Kruskal-Wallis test was used to analyze panel B. ^#^P < .05 and ^##^P < .01 vs control group. *P < .05 and **P < .01 indicate differences within HFHSD-fed groups (fed or not WBE). *B. unif, Bacteroides uniformis* CECT 7771; Bw, body weight; CD, control diet; HFHSD, high-fat high-sugar diet; WBE, wheat bran extract

The expression of il22 in the ileum was increased by the combination of B.uniformis and WBE compared to untreated mice fed CD or HFHSD (Figure 6a). In the small intestine, the abundance of ILC3, a major source of IL22 once activated, was lower in untreated mice fed HFHSD compared to controls and augmented in HFHSD-fed mice receiving *B. uniformis* combined with WBE (Figure 6b). Compared to controls, the detrimental effects of HFHSD on intestinal immunity were related to plasma IL22 reductions (Figure 6c), but not coupled to other changes in plasma pro-inflammatory (IFNɤ and IL12) and anti-inflammatory cytokines (IL10 and IL4) (Figure 6c and Figure S7A). The serum concentration of IFNɤ was lower in HFHSD-fed mice receiving *B. uniformis* combined with WBE and IL22 reduction was attenuated (Figure 6c) compared to untreated mice fed HFHSD, while IL12, IL10, and IL4 remained unchanged (Figure S7A). Expression of *Il22r1* and *Il10r2*, the membrane receptor complex triggering IL22-mediated intracellular cascade, was unaffected in epididymal fat (Figure S7B) while, in liver, *Il10r2* was slightly higher in HFHSD-fed mice receiving *B. uniformis* combined with WBE compared to untreated mice fed HFHSD (Figure 6d), while no changes were observed in *Il22r1* expression. Hepatic IL22 signal transduction tended to be attenuated in untreated mice fed HFHSD as shown by reduced pSTAT3/STAT3 ratio compared to controls (Figure 6e). This ratio was increased by the administration of *B. uniformis* combined with WBE to mice fed HFHSD. HFHSD-feeding altered hepatic inflammation, showing higher levels of IFNɤ and IL10 compared to controls (Figure 6f). Again, *B. uniformis* combined with WBE normalized the increase in both cytokines in liver (Figure 6f), helping to maintain hepatic immune homeostasis in diet-induced obesity. Hepatic levels of IFNɤ or IL10 levels negatively correlated to glycogen levels (Figure 6g).

## Discussion

This study provides a rationale to select dietary fibers that amplifies the benefits of specific intestinal bacteria to tackle obesity, enhancing our knowledge on the underlying mechanisms of action of both the bacterium and the fiber over the immune-metabolic axis. In particular, we demonstrate that body weight and adiposity reduction in diet-induced obesity is greater when *B. uniformis* CECT 7771 and WBE are administered in combination than separately. Furthermore, our study disentangles the respective contribution of *B. uniformis* and WBE to the benefits reported, indicating a key role of the bacterium in regulating oral glucose tolerance, promoting hepatic glucose storage, and influencing selective intestinal lipid absorption and serum triglyceride levels. The study also shows how *B. uniformis* combined with WBE contributes to maintain the intestinal immune homeostasis in obesity, partly by increasing butyrate production, which translates to reduced markers of systemic inflammation and may account for restoration of the metabolic phenotype in diet-induced obesity.

Herein, *B. uniformis* was administered at a lower dose than in previous pre-clinical trials^12^ since we hypothesized its effects would be boosted *in vivo* by its combination with a fiber previously selected as its best carbon source *in vitro*.^16^ Our results confirmed that while this bacterial dose or WBE administered separately failed to induce anti-obesity effects, their combination reduced body weight gain and adiposity. This was accompanied by improvements in energy metabolic routes and restoration of intestinal immune homeostasis. Nevertheless, each intervention separately ameliorated some metabolic alterations in obesity, independently of weight loss; i.e. reduced cholesterolemia and glycemia and improved glucose intolerance, although *B. uniformis* plays a greater role than WBE in restoring metabolic alterations.

The effects of the *B. uniformis*–WBE combination on adiposity were parallel to improvements of some insulin-regulated metabolic routes in adipose tissue and liver. Notably, HFHSD reduced lipogenesis in epididymal fat and hepatic glycogenesis, while both routes were normalized by the *B. uniformis*–WBE combination. In visceral fat, this intervention restored lipogenic markers (*Acc* and *Fas*) and the transcriptional factor regulating their expression *Chrebpα*.^19^ The expression of lipogenic enzymes and *Chrebp* in WAT positively correlates to insulin sensitivity in obesity. In fact, the reactivation of *de novo* lipogenesis in the adipose tissue, which mainly uses glycolysis-derived metabolites as precursors of fatty acids, acts as an insulin sensitizer mechanism improving whole-body metabolism in obese subjects.^20–22^ We also showed hepatic glycogen depots were restored by the *B. uniformis*–WBE combination, which were reduced by HFHSD, supporting a cross-talk between the visceral fat and liver. Furthermore, the combined intervention enhanced both, *Gck* expression, which is insulin-dependent^23^ and its enzymatic product G6P, diminished in mice fed HFHSD. However, this effect was primarily driven by *B. uniformis* as it was also identified in group receiving bacteria alone supporting a major contribution of the bacterium restoring hepatic glycogenesis.

Overall, we identified specific metabolic routes through which the combination of *B. unformis* and WBE favors glucose disposal and improves whole-body insulin sensitivity; i.e. enhancing glucose utilization in the adipocyte of visceral fat to synthesize fatty acids without increasing adiposity and facilitating hepatic glucose storage via stimulation of diverse routes like glucose phosphorylation, which limits glucose output, and glycogenesis.^24^ Targeting these metabolic routes, the *B. uniformis*–WBE combination would maintain physiological lipid storage in adipose tissue to limit ectopic fat accumulation in other organs under a Western diet.

Thermogenesis, mainly fueled by fatty acids,^25^ was increased in HFHSD-fed mice, as indicated by overexpression of *Ucp-1* in BAT and in epididymal fat, whose thermogenic potential may increase under β_3_ adrenergic stimulation.^26^ Although increased adrenergic-induced thermogenesis associated to diet-induced obesity remains controversial,^27^ it may be a mechanism to increase metabolic rate under a Western diet in order to maintain energy balance.^28^ The overexpression of *Ucp-1* in epididymal fat, coupled with increased *Cpt1-a* expression of mice fed HFHSD and their positive correlation to fat mass and weight gain, suggests excess energy from the obesogenic diet was partially counteracted by triggering fatty acid degradation by thermogenesis and fatty acid oxidation, albeit insufficient to prevent obesity. Also, the activation of this mechanism was unnecessary in mice receiving *B. uniformis* and WBE. Therefore, the beneficial effect of *B. uniformis* and WBE on adiposity was not mediated by the activation of energy dissipating metabolic routes using lipids as fuel, which suggests that the improved whole-energy disposal was through a mechanism independent of adipose tissue.

Alternatively, we hypothesized that the *B. uniformis*-WBE combination modifies the metabolites resulting for diet–microbiota interactions, leading to the production of those beneficial for the host metabolism. Notably, WBE increased the concentration of acetate, propionate, and butyrate and the abundance of SCFAs-producing species (i.e. Lachnospiraceae species), which are intestinal bacteria associated with anti-inflammatory properties.^29^ Moreover, the intervention with *B. uniformis* and WBE also led to a gain in several species belonging to the Lachnospiraceae family, which are enriched in oligo- and poly-saccharide degrading genes and SCFA metabolic pathways.^30^ In fact, the *B. uniformis-*WBE combination caused the highest increase in butyrate, one of the key metabolites for improving both metabolism and immunity that could account for the amplified benefits in obesity.

Furthermore, the combined treatment may modify microbial metabolic pathways that, in turn, would modify the nutrients available in the intestinal lumen and thus their accessibility to the host.

Accordingly, and based on the hypothesis that *B. uniformis* combined with WBE would limit the access to HFHSD-associated excess energy by reducing intestinal lipid absorption, we analyzed the impact of each intervention on long-chain FAs, the most abundant dietary component of HFHSD in the cecum, which may indirectly reflect their intestinal uptake. *B. uniformis* combined with WBE increased saturated FAs abundance (stearic and arachidic acids) in the cecum, reflecting their reduced absorption. By contrast, *B. uniformis*, with or without WBE, reduced cecal unsaturated fatty acids (MUFAs, diunsaturated FAs and PUFAs) concentration, which may indicate increased absorption in HFHSD-fed mice. This suggests that *B. uniformis* combined with WBE would attenuate the damaging effects of saturated lipids on the host (i.e. impairment of intestinal integrity, insulin resistance and systemic inflammation). Previous studies support this, demonstrating the metabolic benefits of *B. uniformis* CECT 7771 were associated with reduced fat depots in the enterocytes in diet-induced obese mice.^12^ Moreover, the bacterium with or without WBE would strengthen the metabolic and anti-inflammatory benefits associated with unsaturated FAs. In particular, increased intestinal uptake of oleic acid may improve glucose homeostasis by preventing the impaired β cell function and the inflammatory associated insulin resistance in liver and adipose tissue induced by palmitic acid.^31,32^ In addition, higher absorption of ω3 fatty acids, such as α-linolenic acid, may prevent the disruption of the inflammatory cascade and reverse insulin resistance of diet-induced obese mice.^33^

As the maintenance of the immune homeostasis in the gut protects against systemic inflammation and contributes to preventing onset of obesity and T2D,^34^ we investigated whether this could be a mechanism whereby interventions improved the metabolic phenotype in HFHSD-fed mice. *B. uniformis* and WBE together restored *occludin* expression, a marker of gut integrity, strongly diminished by HFHSD-feeding. *Occludin* expression inversely correlated to weight gain, adiposity and liver glycogen, supporting a relationship between gut integrity and a healthier metabolic state. Furthermore, HFHSD-feeding induced a pro-inflammatory state in the gut showing higher levels of IFNɤ and increased macrophages infiltration into the small intestine. Additionally, we have described for the first time that HFHSD-feeding increased induced IEL and its effector cytokine IFNɤ, while decreasing natural IEL. Each IEL subset mainly diverges in its differentiation process; while naïve T cells differentiate into natural IEL in thymus to directly seed the intestine early in life, induced IEL differentiation occurs in lymphoid intestinal organs in response to local tissue damage.^35,36^ The increased abundance of induced IEL of mice fed HFHSD could result from a response to enhanced bacterial antigen exposure and/or signals of epithelial cell damage under HFHS-feeding, potentially associated with HFHSD-induced deficits in specific microbial species (*Alistipes* spp., *Odoribacter* spp., *Roseburia* spp. and *Christensenella* spp). This, in turn, lowers natural IEL, mainly associated with surveillance and maintenance of intestinal homeostasis.^37,38^ HFHSD also reduced ILC3, which play a critical role in containing commensal bacteria in the luminal side and replenishing epithelial cells in response to a damage^39-40^. Diminished Th17 cells in the lamina propria, the adaptive counterpart of ILC3, accounts for obesity onset by impairing intestinal surveillance which aggravates dysbiosis-mediated inflammation.^41^ Similarly, we also found reduced ILC3 in the intraepithelial layer, the main cells producing IL22 in the intestine.^42^ This suggests that the Western diet diminished the IL22-mediated defense and mucosal reparation mechanisms accounting for the inflammatory state in obesity.^43^ In parallel with the metabolic benefits, the *B. uniformis*–WBE combination completely normalized the abundance of induced IEL and ILC3, both immune cell populations are regulated by the intestinal microbiota and their dietary metabolic products and provide the first line of immune defense against microbes in the gut and their dysregulation causes loss of gut barrier integrity and inflammation.^42,44,45^ Considering gut microbiota analysis, we could postulate that the intervention-mediated restoration of IEL and ILC3 was partly due to the associated changes in gut microbiota composition and derived metabolites like SCFAs which are also present in the small intestine.^46^ SCFAs enhance ILC proliferation^47^ and, in particular, butyrate increases the production of IL22 by RORγt+ ILC3 and CD4 + T cells.^48^ Here we showed that *B.uniformis* combined with WBE restored diversity and enrichment of Lachnospiraceae species that are butyrate producers and induced the higher increase of butyrate in the cecum, mechanisms through which this intervention may promote ILC3 proliferation.

Plasma IL22 was reduced similarly to the intestinal ILC3 in HFHSD-fed mice, suggesting a link between intestinal and systemic immune alterations. We also showed that hepatic IL22 signal transduction cascade was reduced in mice fed HFHSD along with increased IFNɤ and IL10 concentrations, while *B. uniformis* and WBE partially restored IL22 in plasma and IL22 signaling in liver. These findings suggest the combined intervention may attenuate the HFHSD-associated increase in pro-inflammatory IFNɤ-producing cells in liver, causing insulin resistance, through improved IL22 signaling. Supporting our findings, IL22 displays protective functions in response to hepatic tissue damage^49,50^ reducing inflammation in an animal model of alcoholic liver^51^ and improving hepatic insulin sensitivity in obesity.^43,50^

In conclusion, we reveal the mode of action of *B. uniformis* CECT 7771 combined with WBE on the metabolic-immune axis in obesity. Limiting intestinal lipid absorption, lipogenesis-related glucose consumption in the adipose tissue, and glycogenesis in liver seemed to be the metabolic routes through which *B. uniformis* combined with WBE attenuated obesity progression in mice. Furthermore, ILC3 and IEL appeared to be critical intestinal immune mediators of the metabolic benefits of the combined intervention, whose regulation may provide protection against Western diet-induced intestinal immune dysregulation and systemic inflammation.

## Material and methods

### Bacterial growth conditions

*B. uniformis* CECT 7777, a bacterial strain originally isolated from healthy breast-fed infants and deposited in the Spanish Culture Collection (CECT),^53^ was grown in Schaeldler broth at 37°C under anaerobiosis (AnaeroGen, ThermoFisher UK) as previously described.^12,52^ The bacteria were harvested by centrifugation (6000g, 10 min), washed twice with phosphate buffer saline (PBS) solution, and finally re-suspended in PBS containing cysteine (0.05%) and glycerol (10%). Aliquots were immediately frozen in liquid nitrogen and stored at −80°C until use. After freezing and thawing, the number of viable bacteria per milliliter was calculated by counting the colony-forming units (CFU) in Schaeldler agar medium after incubation for 48 h, at 37°C under anaerobic conditions. The bacterial identity was routinely confirmed during production and storage by partial 16S rRNA gene sequencing as described previously.^12^

### Mice and diets

The murine model comprised 6–8 week-old C57BL/6 J male mice (Charles Rivers, France), with *ad-libitum* access to water and food except for oral glucose tolerance test (OGTT), housed in collective cages (5 mice per cage) under 12 h of light/dark cycle (lights on at 8:00) in a temperature-controlled room (23 ± 2°C). Mice were randomly allocated into 5 experimental groups by the animal healthcare technicians. For 17 weeks, mice were fed either control diet (CD; D12450K Research Diet NJ, USA; 10% of energy from fat and without sucrose; N = 10), high-fat high-sugar diet (HFHSD, D12451 Research Diet NJ, USA; 45% of energy from fat and 35% from sucrose; N = 20) or HFHSD supplemented with 5% of wheat-bran extract (WBE, provided by Cargill, Belgium) (HFHSD-WBE; N = 20) (dietary FAs composition is detailed in Table S1). Both mice fed HFHSD alone or HFHSD+WBE were subdivided into two experimental groups. One of these groups received an oral dose of vehicle (PBS with 0.05% cysteine and 10% glycerol, N = 10) and the other an oral dose *B. uniformis* CECT 7771 (5x10^7^ CFU per mouse, N = 10) daily. CD-fed mice received only vehicle (N = 10).

Body weight was determined weekly. Glucose, triglycerides, and cholesterol in plasma were measured at week 12 and an oral glucose tolerance test (OGTT) was conducted at week 14. At week 17, cardiac puncture was performed in isoflurane anesthetized mice for blood collection. Animals were then sacrificed by cervical dislocation and liver, epididymal white adipose tissue (WAT), ileum and colon (2 cm pieces both) tissues and cecal content were isolated, snap-frozen in liquid nitrogen and stored at −80°C until use. Whole small intestine (except the 2 cm-long distal part) was embedded in cold PBS and immediately processed for the analysis of intraepithelial mononuclear cells by flow cytometry. Another batch of animals were used to estimate food efficiency at week 6 of HFHSD intervention by calculating the ratio between the weight gain and the amount of energy consumed *ad libitum* during 24 h after 18 h of fasting (refeeding period) (experimental procedure detailed in Figure S1). All experimental procedures were performed in accordance with European Union 2010/63/UE and Spanish RD53/2013 guidelines and approved by the ethics committee of the University of Valencia (Animal Production Section, Central Service of Support to Research [SCSIE], University of Valencia, Spain) and authorized by Dirección General de Agricultura, Ganadería y Pesca (Generalidad Valenciana) (approval ID 2018/VSC/PEA/0090).

### Oral glucose tolerance test (OGTT)

At week 14, an OGTT was conducted in 4-hour fasted mice. Blood from the saphenous vein was collected at 0, 15, 30, 60, and 120 minutes of being administered an oral glucose load (2 g/Kg). Glucose was measured through glucose test strips using a glucometer (CONTOUR®-NEXT meter, Bayer, Leverkusen, Germany).

### Blood metabolic parameters

Blood from the mandibular vein of 4-hour fasted mice was collected at week 12 in microtubes with K3 EDTA (Sarstedt, Nümbrecht Germany) to determine glycemia, measured as conducted in the OGTT, and triglycerides and cholesterol in plasma (blood centrifugation at 400 g for 15 minutes at 4°C) using a colorimetric kit assay according to manufacturer´s instructions (Química Clínica Aplicada, Tarragona, Spain). Plasma from blood collected by cardiac puncture in 4-hour fasted mice at week 17 was used to measure insulin, glucagon-like peptide 1 (GLP-1), peptide YY (PYY) and the cytokine tumor necrosis factor α (TNFα), interleukins IL12, IL10, IL4, and IL22 and interferon ɤ (IFNɤ) using Multiplex Assays Using Luminex® (Milliplex, Merck group, Darmstadt, Germany) according to manufacturer´s instructions.

### Isolation of intraepithelial mononuclear cells and flow cytometry analysis

Isolation of intraepithelial cells was conducted as previously described.^54,55^ In brief, small intestine was washed with cold PBS and longitudinally opened and cut into small pieces. For the isolation of the epithelium, tissue was incubated twice in Hansk´s balanced salt solution with calcium and magnesium (HBSS; ThermoFisher Scientific, Massachusetts, USA) containing 5 mM EDTA (Scharlab), 1 mM DTT, 100 µg/ml streptomycin and 100 U/ml penicillin (Merck, Darmstadt, Germany) for 30 minutes at 37°C with orbital shaking. After each incubation, supernatant fractions were filtrated using 100 µm nylon cell strainers (Biologix, Shandong, China) and centrifuged to harvest cell suspensions. Remaining tissue was washed with PBS for being incubated twice with HBSS containing 0.5 mg/mL collagenase D (Roche Diagnostics GmbH, Mannheim, Germany), 3 mg/mL dispase II (Sigma‐Aldrich), 1 mg/mL DNase I (Roche Diagnostics GmbH), and streptomycin 100 µg/ml and penicillin 100 U/ml with orbital agitation for 30 minutes at 37°C. Cells from the lamina propria were collected by filtrating supernatant fractions with 70 µm nylon cell strainers that were then centrifuged to harvest cell suspensions. Isolated cells in FACS buffer (PBS with BSA 0.5%) were then incubated with different immune markers during 30 min at 4°C in darkness to measure lymphocyte subpopulations (natural and induced) in the epithelium as follows. Natural (CD45+ CD2+ CD5+) and induced (CD45+ CD2- CD5-) intraepithelial lymphocytes (IEL) were determined by phycoerithrin (PE)-conjugated anti-CD45, allophycocyanin (APC)-conjugated anti-CD2 and PE-Vio770-conjugated anti-CD5 (Miltenyi Biotec, Bergisch Gladbach, Germany) antibodies. Innate lymphoid cells (ILC) type 1 (ILC1: CD90+ CD127+ LIN- Tbet+ IFNɤ+) and type 3 (ILC3: CD90+ CD127+ LIN- RORɤt+ IL22+) were labeled by PerCP-Cy™5.5-conjugated anti-Lineage antibody cocktail (LIN) (BD-Bioscience, USA), phycoerithrin (PE)-conjugated anti-Tbet, allophycocyanin (APC)-conjugated anti-IFNɤ, phycoerithrin (PE)-conjugated anti- RORɤt (Miltenyi Biotec, Bergisch Gladbach, Germany) and allophycocyanin (APC)-conjugated anti-IL22 (Biolegend, San Diego, California, USA) antibodies. Pro-inflammatory (M1: F4/80+ CD80+ iNOS+) and anti-inflammatory (M2: F4/80+ CD206+ Arg1+) macrophages from lamina propria were determined using FITC-conjugated antiF4/80+, Pe-Vio770-conjugated anti-CD80 (Miltenyi, Biotec, Bergisch Gladbach, Germany), APC-conjugated anti-iNOS (Thermo Fisher Scientific, MA, USA), PerCPCy5.5-conjugated anti-CD206 (Biolegend, San Diego, California, USA) and PE-conjugated anti-Arg1 (R&D Systems, Minneapolis, USA) antibodies. Additionally, for intracellular markers staining (Tbet, IFNɤ, RORɤt, IL22, iNOS, and Arg1) cells were permeabilized and fixed (fixation/permeabilization solution kit, BD Bioscience, USA). Data acquisition and analysis were performed using a BD LSRFortessa (Becton Dickinson, USA) flow cytometer operated by FACS Diva software v.7.0 (BD Biosciences, USA).

### Gene expression by RT-qPCR

Total RNA was isolated with TRIsure^TM^ reagent (Bioline, London, UK) according to manufacturer´s instructions. For reverse transcription to cDNA, 2–1.5 μg of RNA were incubated with reverse transcription buffer, dNTPs (4 mM), random primers, and 50 units of MultiScribe^TM^ Reverse Transcriptase at 25°C for 10 minutes followed by 120 minutes at 37°C and 5 minutes at 85°C. The qPCR was performed on a LightCycler® 480 Instrument (Roche, Boulogne-Billancourt, France). The reaction consisted of LightCycler 480 SYBR Green I Master mix (Roche, Branchburg, USA) and 300 nM of gene-specific primer pairs. The primer sequences of each gene are detailed in **Appendix Table S2**. Ribosomal protein L19 (Rpl19) was used as housekeeping gene. The qPCR program included the following steps: a denaturation step at 95°C for 10 minutes; followed by 45 amplification cycles (95°C for 10 seconds, 60°C for 30 seconds, and 72°C for 5 seconds) and a final melting stage at 95°C for 5 seconds and at 65°C for 1 minute. Variation of cDNA abundance was calculated according to the 2^−(ΔΔCt)^ method and represented as fold change expression relative to the control group.

### Western blot

One hundred micrograms of denatured total proteins from liver, extracted using RIPA buffer (Merck, Germany) were separated by SDS-PAGE electrophoresis and transferred onto polyvinylidene difluoride (PVDF) membranes (Thermo Fisher Scientific, MA, USA). Membranes were overnight incubated at 4°C with 1:1000 dilution of phospho-STAT3 (Tyr705, #9138 Cell Signaling, Beverly, MA, USA), STAT3 (#4904 Cell Signaling, Beverly, MA, USA) and β-actin (#4967 Cell Signaling, Beverly, MA, USA) primary antibodies followed by 1 h incubation at RT with horse anti-mouse IgG-HRP at 1:5000 (#7076 Cell Signaling, Beverly, MA, USA) for phospho-STAT3 antibody binding and goat anti-rabbit IgG-HRP at 1:5000 (#7074 Cell Signaling, Beverly, MA, USA) for STAT3 and β-actin antibodies binding. Chemiluminescence signal was enhanced by adding ECL (SuperSignalTM West Dura Extended Duration Substrate, Thermo Fisher Scientific, MA, USA) and quantified using ImageJ 1.8 software.

### Metabolites and cytokines in liver

Lipids from liver were extracted as previously described.^56,57^ In brief, a solution of chloroform/methanol (2:1) was used for tissue homogenization. After 3 h of shaking, milliQ water was added and the organic layer was separated by centrifugation (16000 g, 20 minutes), collected, and dried overnight. Then, triglycerides and free fatty acids were measured in the isolated organic layer using the Triglyceride colorimetric Assay Kit (Elabscience, USA) and the Free Fatty Acid Quantitation Kit (Sigma, Missouri, USA), respectively. Hepatic glycogen and glucose-6-phosphate (G6P) were quantified in the total homogenized tissue through the Glycogen Assay Kit (Sigma-Aldrich, Missouri, USA) and the Glucose-6-Phosphate Colorimetric Assay Kit (BioVision, California, USA), respectively. Cytokines (TNFα, IL10, IL6, and IFNɤ) were measured using ProcartaPlex multiplex immunoassays according to manufacturer´s instructions (Thermo Fisher Scientific, MA, USA)

### Fatty acid analysis

Cecal content was homogenized by bead beating in 70%-isopropanol.^58^ SCFA were quantified by liquid chromatography coupled to tandem mass spectrometry (LC-MS/MS) upon derivatization to 3-nitrophenylhydrazones.^58^ Concentrations of total fatty acids were determined by gas chromatography coupled to mass spectrometry (GC-MS) after derivatization to fatty acid methyl esters (FAMEs).^59^

### Microbiota analysis

DNA of the cecal content was isolated using the QIAamp PowerFecal DNA kit (Qiagen, Hilden, Germany) following the manufacturer’s instructions. The DNA concentration was measured by UV methods (Nanodrop, Thermo Scientific, Wilmington, USA) and an aliquot of every sample was prepared at ~20 ng/µL with nuclease-free water for polymerase chain reaction (PCR). The V3-V4 hypervariable regions of the 16S ribosomal ribonucleic acid (rRNA) gene were amplified using 1 μL aliquot DNA and 25 PCR cycles consisting of the following steps: 95°C for 20 sec., 55°C for 20 sec., and 72°C for 20 sec. Phusion High-Fidelity Taq Polymerase (Thermo Scientific, Wilmington, USA) and the 6-mer barcoded primers, S-D-Bact-0341-b-S-17 (TAGCCTACGGGNGGCWGCAG) and S-D-Bact-0785-a-A-21 (ACTGACTACHVGGGTATCTAATCC), which target a wide repertoire of bacterial 16S rRNA genes,^60^ were used for PCR. Dual-barcoded PCR products, consisting of ~500 bp, were purified from triplicate reactions with the Illustra GFX PCR DNA and Gel Band Purification Kit (GE Healthcare, UK) and quantified through Qubit 3.0 and the Qubit dsDNA HS Assay Kit (Thermo Fisher Scientific, Waltham, MA, USA). The samples were multiplexed in one sequencing run by combining equimolar quantities of amplicon DNA (~50 ng per sample) and sequenced in one lane of the Illumina MiSeq platform with 2 × 300 PE configuration (Eurofins Genomics GmbH, Germany). Raw data were delivered in fastq files and pair ends with quality filtering were assembled using Flash software.^61^ Sample de-multiplexing was carried out using sequence information from forward/reverse primers, the respective DNA barcodes, and the SeqKit suite of analysis.^62^ After demultiplexing, the barcodes/primers were removed and sequences were processed for chimera removal using UCHIME algorithm^63^ and the SILVA reference set of 16S sequences (Release 128).^64^ Operational Taxonomic Unit (OTU) information was retrieved by using a rarefied subset of 17,000 sequences per sample, randomly selected after multiple shuffling (10,000X) from the original dataset, and the UCLUST algorithm implemented in USEARCH v8.0.1623.^65^ Common alpha diversity descriptors including the Observed OTUs, Reciprocal Simpson’s index, dominance, and phylogenetic distance (PD) were computed using QIIME v1.9.1.^66^ For phylogenetic-based metrics, the OTUs sequences were aligned using the PyNAST algorithm implemented in QIIME and the SILVA aligned reference database, whereas the tree topology reconstruction was completed with the FastTree algorithm^67^ using the generalized time-reversible (GTR) model and gamma-based likelihood. The evaluation of the community structure across the sample groups was performed with the *Vegan* R package through interpretative multivariate and constrained appraisal based on the distance-based redundancy analysis (dbRDA) (*vegan::dbrda* function and “bray” method). Taxonomic identification of differentially abundant OTUs was achieved by submitting respective sequences to the SINA aligner web server (https://www.arb-silva.de/aligner/) and using the SILVA database for classification.

### Statistical analysis

G*Power 3.1.9.2 was used to calculate sample size and SPSS (IBM SPSS Statistics 24) to conduct statistics. Shapiro–Wilk test was employed to assess normality. Statistics of normal distributed data were performed using different parametric tests, including repeated measures two-way ANOVA for the analysis of multiple measures of the same variable taken on the same subjects over the time (body weight gain and OGTT); one-way ANOVA followed by post-hoc Tuckey test to compare controls (CD-fed mice orally administered the vehicle) vs HFHSD-fed mice, receiving or not the bacterium and/or WBE and two-way ANOVA restricted to HFHSD-fed mice to examine both, the main effect of each independent variable (*B. uniformis* or the WBE) and the interactions between them. For the analysis showing interactions between variables a post-hoc Tukey test was conducted. Kruskal–Wallis test followed by pairwise multiple comparisons was used to analyze non-normally distributed data. Bravais-Pearson´s correlation coefficient was used to test correlations between two variables. Only set of data with not significantly different slopes and intercepts, analyzed using linear regression, were combined to calculate a global correlation coefficient between variables.

Statistical analyses on microbiota data were done applying non-parametric methods such as Kruskal–Wallis and pairwise Wilcoxon Rank Sum tests (for unpaired samples) with Benjamini-Hochberg *post hoc* correction for multiple group comparison across the alpha diversity descriptors. Statistical differences in the microbial community structure were assessed by the permutation-based *vegan::adonis* function. Differential abundance of OTUs across groups was assisted by applying the Kruskal–Wallis test with Benjamini-Hochberg *post hoc* correction. Highly divergent OTUs in terms of abundance were selected when Kruskal–Wallis chi-squared test was ≥30 and corrected P ≤ 0.001. Additionally, pairwise Wilcoxon Rank Sum test with Benjamini-Hochberg correction was applied to identify precise sample groups showing up- or down-representation of different OTUs. Graphs and plots were drawn using *ggplot2* and *grid* R v3.6 packages, Heatmap hierarchical clustering of OTUs (scaled read counts) was obtained using *“correlation”* as a distance and “*complete”* as clustering method. Results were not assessed by blinded investigators.

## Supplementary Material

Supplemental Material

## Data Availability

The caecal content microbiota data generated in this study is publicly available in the European Nucleotide Archive (ENA), and the raw fastq files associated can be accessed through the bioproject accession number PRJEB37867 https://www.ebi.ac.uk/ena/data/search?query=PRJEB37867

## Abbreviations

*Acc*, acetyl-CoA carboxylase

AXOS arabinoxylan oligosaccharides

AUC, area under the curve

BAT, brown adipose tissue

*Chrebpα*, carbohydrate responsive-element-binding protein α

*Cpt1a*, carnitine palmitoyltransferase 1a

*Fas*, fatty acid synthase; FAs, fatty acids

GLP-1, glucagon-like peptide-1

*Gck*, glucokinase

*Glut2*, glucose transporter 2

*Glut4*, glucose transporter 4

G6P, glucose-6-phosphate

HFHSD, high-fat high-sugar diet

*Hsl*, hormone sensitive lipase

ILC, innate lymphoid cells

IEL, intraepithelial lymphocytes

*Lpl*, lipoprotein lipase

*Lyz1*, lysozyme 1

MUFAs, monounsaturated Fas

OGTT, oral glucose tolerance test

PYY, peptide YY *Pla2g2a*, phospholipase A2 group IIA

PUFAs, polyunsaturated Fas

*Reg3ɤ*, regenerating islet-derived protein 3 gamma

SCFAs, short-chain fatty acids

T2D, type-2 diabetes

*Ucp-1*, uncoupling protein-1

WBE, wheat-bran extract

WAT, white adipose tissue.

## References

[1] Cani PD. Human gut microbiome: hopes, threats and promises. Gut. 2018;67(9):1716–1725. doi:10.1136/gutjnl-2018-316723. 29934437 PMC6109275

[2] Rooks MG, Garrett WS. Gut microbiota, metabolites and host immunity. Nat Rev Immunol. 2016;16(6):341–352. doi:10.1038/nri.2016.42. 27231050 PMC5541232

[3] Hooper LV, Littman DR, Macpherson AJ. Interactions between the microbiota and the immune system. Science. 2012;336(6086):1268–1273. doi:10.1126/science.1223490. 22674334 PMC4420145

[4] Sonnenburg ED, Smits SA, Tikhonov M, Higginbottom SK, Wingreen NS, Sonnenburg JL. Diet-induced extinctions in the gut microbiota compound over generations. Nature. 2016;529(7585):212–215. doi:10.1038/nature16504. 26762459 PMC4850918

[5] Wan Y, Wang F, Yuan J, Li J, Jiang D, Zhang J, Li H, Wang R, Tang J, Huang T, et al. Effects of dietary fat on gut microbiota and faecal metabolites, and their relationship with cardiometabolic risk factors: a 6-month randomised controlled-feeding trial. Gut. 2019;68(8):1417–1429. doi:10.1136/gutjnl-2018-317609. 30782617

[6] Levy M, Thaiss CA, Zeevi D, Dohnalová L, Zilberman-Schapira G, Mahdi JA, David E, Savidor A, Korem T, Herzig Y, et al. Microbiota-modulated metabolites shape the intestinal microenvironment by regulating nlrp6 inflammasome signaling. Cell. 2015;163(6):1428–1443. doi:10.1016/j.cell.2015.10.048. 26638072 PMC5665753

[7] Caesar R, Tremaroli V, Kovatcheva-Datchary P, Cani PD, Bäckhed F. Crosstalk between gut microbiota and dietary lipids aggravates WAT inflammation through TLR signaling. Cell Metab. 2015;22(4):658–668. doi:10.1016/j.cmet.2015.07.026. 26321659 PMC4598654

[8] Tilg H, Zmora N, Adolph TE, Elinav E. The intestinal microbiota fuelling metabolic inflammation. Nat Rev Immunol. 2020;20(1):40–54. doi:10.1038/s41577-019-0198-4. 31388093

[9] Rodriguez J, Hiel S, Neyrinck AM, Le Roy T, Pötgens SA, Leyrolle Q, Pachikian BD, Gianfrancesco MA, Cani PD, Paquot N, et al. Discovery of the gut microbial signature driving the efficacy of prebiotic intervention in obese patients. Gut. 2020;69(11). doi:10.1136/gutjnl-2019-319726.. PMC7569399 32041744

[10] Adeshirlarijaney A, Gewirtz AT. Considering gut microbiota in treatment of type 2 diabetes mellitus. Gut Microbes. 2020;11(3):253–264. doi:10.1080/19490976.2020.1717719. 32005089 PMC7524291

[11] Romaní-Pérez M, Agusti A, Sanz Y. Innovation in microbiome-based strategies for promoting metabolic health. Curr Opin Clin Nutr Metab Care. 2017;20(6):484–491. doi:10.1097/MCO.0000000000000419. 28862999

[12] Gauffin Cano P, Santacruz A, Moya Á, Sanz Y. Bacteroides uniformis CECT 7771 ameliorates metabolic and immunological dysfunction in mice with high-fat-diet induced obesity. PLoS ONE. 2012;7(7):e41079. doi:10.1371/journal.pone.0041079. 22844426 PMC3406031

[13] Delzenne NM, Olivares M, Neyrinck AM, Beaumont M, Kjølbæk L, Larsen TM, Benítez-Páez A, Romaní-Pérez M, Garcia-Campayo V, Bosscher D, et al . Nutritional interest of dietary fiber and prebiotics in obesity: lessons from the MyNewGut consortium. Clin Nutr. 2019. doi:10.1016/j.clnu.2019.03.002. 30904186

[14] Portune KJ, Benítez-Páez A, Del Pulgar EMG, Cerrudo V, Sanz Y. Gut microbiota, diet, and obesity-related disorders-The good, the bad, and the future challenges. Mol Nutr Food Res. 2017;61(1). doi:10.1002/mnfr.201600252. 27287778

[15] EFSA Panel on Dietetic Products, Nutrition, and Allergies (NDA). Scientific opinion on dietary reference values for carbohydrates and dietary fibre. EFSA J. 2010;3(8):1462.

[16] Benítez-Páez A, Gómez Del Pulgar EM, Sanz Y. The glycolytic versatility of bacteroides uniformis CECT 7771 and its genome response to oligo and polysaccharides. Front Cell Infect Microbiol. 2017;7:383. doi:10.3389/fcimb.2017.00383. 28971068 PMC5609589

[17] Boll EVJ, Ekström LMNK, Courtin CM, Delcour JA, Nilsson AC, Björck IME, Östman EM. Effects of wheat bran extract rich in arabinoxylan oligosaccharides and resistant starch on overnight glucose tolerance and markers of gut fermentation in healthy young adults. Eur J Nutr. 2016;55(4):1661–1670. doi:10.1007/s00394-015-0985-z. 26169871

[18] Kjølbæk L, Benítez-Páez A, Gómez Del Pulgar EM, Brahe LK, Liebisch G, Matysik S, Rampelli S, Vermeiren J, Brigidi P, Larsen LH, et al. Arabinoxylan oligosaccharides and polyunsaturated fatty acid effects on gut microbiota and metabolic markers in overweight individuals with signs of metabolic syndrome: A randomized cross-over trial. Clin Nutr. 2020;39(1):67–79. doi:10.1016/j.clnu.2019.01.012. 30827722

[19] Iizuka K, Bruick RK, Liang G, Horton JD, Uyeda K. Deficiency of carbohydrate response element-binding protein (ChREBP) reduces lipogenesis as well as glycolysis. Proc Natl Acad Sci U S A. 2004;101(19):7281–7286. doi:10.1073/pnas.0401516101. 15118080 PMC409910

[20] Herman MA, Peroni OD, Villoria J, Schön MR, Abumrad NA, Blüher M, Klein S, Kahn BB. A novel ChREBP isoform in adipose tissue regulates systemic glucose metabolism. Nature. 2012;484(7394):333–338. doi:10.1038/nature10986. 22466288 PMC3341994

[21] Eissing L, Scherer T, Tödter K, Knippschild U, Greve JW, Buurman WA, Pinnschmidt HO, Rensen SS, Wolf AM, Bartelt A, et al. De novo lipogenesis in human fat and liver is linked to ChREBP-β and metabolic health. Nat Commun. 2013;4(1):1528. doi:10.1038/ncomms2537. 23443556 PMC3740744

[22] Solinas G, Borén J, Dulloo AG. De novo lipogenesis in metabolic homeostasis: more friend than foe? Mol Metab. 2015;4(5):367–377. doi:10.1016/j.molmet.2015.03.004. 25973385 PMC4421107

[23] Petersen MC, Vatner DF, Shulman GI. Regulation of hepatic glucose metabolism in health and disease. Nat Rev Endocrinol. 2017;13(10):572–587. doi:10.1038/nrendo.2017.80. 28731034 PMC5777172

[24] Rui L. Energy metabolism in the liver. Compr Physiol. 2014;4:177–197. 24692138 10.1002/cphy.c130024 PMC4050641

[25] Chouchani ET, Kazak L, Spiegelman BM. New advances in adaptive thermogenesis: UCP1 and beyond. Cell Metab. 2019;29(1):27–37. doi:10.1016/j.cmet.2018.11.002. 30503034

[26] Zhang F, Hao G, Shao M, Nham K, An Y, Wang Q, Zhu Y, Kusminski CM, Hassan G, Gupta RK, et al. An adipose tissue atlas: an image-guided identification of human-like BAT and beige depots in rodents. Cell Metab. 2018;27(1):252–262.e3. doi:10.1016/j.cmet.2017.12.004. 29320705 PMC5764189

[27] Kozak LP. Brown fat and the myth of diet-induced thermogenesis. Cell Metab. 2010;11(4):263–267. doi:10.1016/j.cmet.2010.03.009. 20374958 PMC2867325

[28] Feldmann HM, Golozoubova V, Cannon B, Nedergaard J. UCP1 ablation induces obesity and abolishes diet-induced thermogenesis in mice exempt from thermal stress by living at thermoneutrality. Cell Metab. 2009;9(2):203–209. doi:10.1016/j.cmet.2008.12.014. 19187776

[29] Hu S, Wang J, Xu Y, Yang H, Wang J, Xue C, Yan X, Su L. Anti-inflammation effects of fucosylated chondroitin sulphate from acaudina molpadioides by altering gut microbiota in obese mice. Food Funct. 2019;10(3):1736–1746. doi:10.1039/C8FO02364F. 30855043

[30] Vacca M, Celano G, Calabrese FM, Portincasa P, Gobbetti M, De Angelis M. The controversial role of human gut Lachnospiraceae. Microorganisms. 2020;8(4). doi:10.3390/microorganisms8040573. PMC7232163 32326636

[31] Maedler K, Oberholzer J, Bucher P, Spinas GA, Donath MY. Monounsaturated fatty acids prevent the deleterious effects of palmitate and high glucose on human pancreatic beta-cell turnover and function. Diabetes. 2003;52(3):726–733. doi:10.2337/diabetes.52.3.726. 12606514

[32] Palomer X, Pizarro-Delgado J, Barroso E, Vázquez-Carrera M. Palmitic and oleic acid: the yin and yang of fatty acids in type 2 diabetes mellitus. Trends Endocrinol Metab. 2018;29(3):178–190. doi:10.1016/j.tem.2017.11.009. 29290500

[33] Oliveira V, *et al*. Oliveira V, Marinho R, Vitorino D, Santos GA, Moraes JC, Dragano N, Sartori-Cintra A, Pereira L, Catharino RR, da Silva ASR, et al. Diets containing α-linolenic (ω3) or oleic (ω9) fatty acids rescues obese mice from insulin resistance. Endocrinology. 2015;156(11):4033–4046. doi:10.1210/en.2014-1880. 26280128

[34] Winer DA, Luck H, Tsai S, Winer S. The intestinal immune system in obesity and insulin resistance. Cell Metab. 2016;23(3):413–426. doi:10.1016/j.cmet.2016.01.003. 26853748

[35] Mayassi T, Jabri B. Human intraepithelial lymphocytes. Mucosal Immunol. 2018;11(5):1281–1289. doi:10.1038/s41385-018-0016-5. 29674648 PMC6178824

[36] Cheroutre H, Lambolez F, Mucida D. The light and dark sides of intestinal intraepithelial lymphocytes. Nat Rev Immunol. 2011;11(7):445–456. doi:10.1038/nri3007. 21681197 PMC3140792

[37] Boismenu R, Havran WL. Modulation of epithelial cell growth by intraepithelial gamma delta T cells. Science. 1994;266:1253–1255. doi:10.1126/science.7973709. 7973709

[38] Roberts SJ, Smith AL, West AB, Wen L, Findly RC, Owen MJ, Hayday AC. T-cell alpha beta + and gamma delta + deficient mice display abnormal but distinct phenotypes toward a natural, widespread infection of the intestinal epithelium. Proceedings of the National Academy of Sciences. 1996;93(21):11774–11779. doi:10.1073/pnas.93.21.11774. PMC38134 8876213

[39] Ebbo M, Crinier A, Vély F, Vivier E. Innate lymphoid cells: major players in inflammatory diseases. Nat Rev Immunol. 2017;17(11):665–678. doi:10.1038/nri.2017.86. 28804130

[40] Omenetti S, Bussi C, Metidji A, Iseppon A, Lee S, Tolaini M, Li Y, Kelly G, Chakravarty P, Shoaie S, et al. The intestine harbors functionally distinct homeostatic tissue-resident and inflammatory Th17 Cells. Immunity. 2019;51(1):77–89.e6. doi:10.1016/j.immuni.2019.05.004. 31229354 PMC6642154

[41] Garidou L, Pomié C, Klopp P, Waget A, Charpentier J, Aloulou M, Giry A, Serino M, Stenman L, Lahtinen S, et al. The gut microbiota regulates intestinal CD4 T cells expressing rorγt and controls metabolic disease. Cell Metab. 2015;22(1):100–112. doi:10.1016/j.cmet.2015.06.001. 26154056

[42] Sawa S, Lochner M, Satoh-Takayama N, Dulauroy S, Bérard M, Kleinschek M, Cua D, Di Santo JP, Eberl G. RORγt+ innate lymphoid cells regulate intestinal homeostasis by integrating negative signals from the symbiotic microbiota. Nat Immunol. 2011;12(4):320–326. doi:10.1038/ni.2002. 21336274

[43] Wang X, Ota N, Manzanillo P, Kates L, Zavala-Solorio J, Eidenschenk C, Zhang J, Lesch J, Lee WP, Ross J, et al. Interleukin-22 alleviates metabolic disorders and restores mucosal immunity in diabetes. Nature. 2014;514(7521):237–241. doi:10.1038/nature13564. 25119041

[44] Ismail AS, Severson KM, Vaishnava S, Behrendt CL, Yu X, Benjamin JL, Ruhn KA, Hou B, DeFranco AL, Yarovinsky F, et al. Gammadelta intraepithelial lymphocytes are essential mediators of host-microbial homeostasis at the intestinal mucosal surface. Proc Natl Acad Sci U S A. 2011;108(21):8743–8748. doi:10.1073/pnas.1019574108. 21555560 PMC3102410

[45] McDonald BD, Jabri B, Bendelac A. Diverse developmental pathways of intestinal intraepithelial lymphocytes. Nat Rev Immunol. 2018;18(8):514–525. doi:10.1038/s41577-018-0013-7. 29717233 PMC6063796

[46] Cummings JH, Pomare EW, Branch WJ, Naylor CP, Macfarlane GT. Short chain fatty acids in human large intestine, portal, hepatic and venous blood. Gut. 1987;28(10):1221–1227. doi:10.1136/gut.28.10.1221. 3678950 PMC1433442

[47] Sepahi A, Liu Q, Friesen L, Kim CH. Dietary fiber metabolites regulate innate lymphoid cell responses. Mucosal Immunol. 2020. doi:10.1038/s41385-020-0312-8.. PMC7736174 32541842

[48] Yang W, Yu T, Huang X, Bilotta AJ, Xu L, Lu Y, Sun J, Pan F, Zhou J, Zhang W, et al. Intestinal microbiota-derived short-chain fatty acids regulation of immune cell IL-22 production and gut immunity. Nat Commun. 2020;11(1):4457. doi:10.1038/s41467-020-18262-6. 32901017 PMC7478978

[49] Zenewicz LA, Yancopoulos GD, Valenzuela DM, Murphy AJ, Karow M, Flavell RA. Interleukin-22 but not interleukin-17 provides protection to hepatocytes during acute liver inflammation. Immunity. 2007;27(4):647–659. doi:10.1016/j.immuni.2007.07.023. 17919941 PMC2149911

[50] Sabat R, Wolk K. Deciphering the role of interleukin-22 in metabolic alterations. Cell Biosci. 2015;5(1):68. doi:10.1186/s13578-015-0060-8. 26674616 PMC4678716

[51] Hendrikx T, Duan Y, Wang Y, Oh J-H, Alexander LM, Huang W, Stärkel P, Ho SB, Gao B, Fiehn O, et al. Bacteria engineered to produce IL-22 in intestine induce expression of REG3G to reduce ethanol-induced liver disease in mice. Gut. 2019;68(8):1504–1515. doi:10.1136/gutjnl-2018-317232. 30448775 PMC6387784

[52] Sánchez E, De Palma G, Capilla A, Nova E, Pozo T, Castillejo G, Varea V, Marcos A, Garrote JA, Polanco I, et al. Influence of environmental and genetic factors linked to celiac disease risk on infant gut colonization by Bacteroides species. Appl Environ Microbiol. 2011;77(15):5316–5323. doi:10.1128/AEM.00365-11. 21642397 PMC3147488

[53] Gómez Del Pulgar EM, Benítez-Páez A, Sanz Y. Safety assessment of bacteroides uniformis CECT 7771, a symbiont of the gut microbiota in infants. Nutrients. 2020;12(2). doi:10.3390/nu12020551. PMC7071458 32093252

[54] Juanola O, Piñero P, Gómez-Hurtado I, Caparrós E, García-Villalba R, Marín A, Zapater P, Tarín F, González-Navajas JM, Tomás-Barberán FA, et al. Regulatory T cells restrict permeability to bacterial antigen translocation and preserve short-chain fatty acids in experimental cirrhosis. Hepatol Commun. 2018;2(12):1610–1623. doi:10.1002/hep4.1268. 30556045 PMC6287488

[55] Moratalla A, Gómez-Hurtado I, Moya-Pérez Á, Zapater P, Peiró G, González-Navajas JM, Gómez Del Pulgar EM, Such J, Sanz Y, Francés R. Bifidobacterium pseudocatenulatum CECT7765 promotes a TLR2-dependent anti-inflammatory response in intestinal lymphocytes from mice with cirrhosis. Eur J Nutr. 2016;55(1):197–206. doi:10.1007/s00394-015-0837-x. 25657013

[56] Storlien LH, *et al*. Influence of dietary fat composition on development of insulin resistance in rats. relationship to muscle triglyceride and omega-3 fatty acids in muscle phospholipid. Diabetes. 1991;40(2):280–289. doi:10.2337/diab.40.2.280. 1991575

[57] Frayn KN, Maycock PF. Skeletal muscle triacylglycerol in the rat: methods for sampling and measurement, and studies of biological variability. J Lipid Res. 1980;21:139–144. 7354251

[58] Liebisch G, Ecker J, Roth S, Schweizer S, Öttl V, Schött H-F, Yoon H, Haller D, Holler E, Burkhardt R, et al. Quantification of fecal short chain fatty acids by liquid chromatography tandem mass spectrometry-investigation of pre-analytic stability. Biomolecules. 2019;9(4). doi:10.3390/biom9040121. PMC6523859 30925749

[59] Ecker J, Scherer M, Schmitz G, Liebisch G. A rapid GC-MS method for quantification of positional and geometric isomers of fatty acid methyl esters. J Chromatogr B Analyt Technol Biomed Life Sci. 2012;897:98–104. doi:10.1016/j.jchromb.2012.04.015. 22542399

[60] Klindworth A, Pruesse E, Schweer T, Peplies J, Quast C, Horn M, Glöckner FO. Evaluation of general 16S ribosomal RNA gene PCR primers for classical and next-generation sequencing-based diversity studies. Nucleic Acids Res. 2013;41(1):e1. doi:10.1093/nar/gks808. 22933715 PMC3592464

[61] Magoč T, Salzberg SL. FLASH: fast length adjustment of short reads to improve genome assemblies. Bioinformatics. 2011;27(21):2957–2963. doi:10.1093/bioinformatics/btr507. 21903629 PMC3198573

[62] Shen W, Le S, Li Y, Hu F. SeqKit: a cross-platform and ultrafast toolkit for FASTA/Q file manipulation. PLoS ONE. 2016;11(10):e0163962. doi:10.1371/journal.pone.0163962. 27706213 PMC5051824

[63] Edgar RC, Haas BJ, Clemente JC, Quince C, Knight R. UCHIME improves sensitivity and speed of chimera detection. Bioinformatics. 2011;27(16):2194–2200. doi:10.1093/bioinformatics/btr381. 21700674 PMC3150044

[64] Quast C, Pruesse E, Yilmaz P, Gerken J, Schweer T, Yarza P, Peplies J, Glöckner FO. The SILVA ribosomal RNA gene database project: improved data processing and web-based tools. Nucleic Acids Res. 2013;41(D1):D590–596. doi:10.1093/nar/gks1219. 23193283 PMC3531112

[65] Edgar RC. Search and clustering orders of magnitude faster than BLAST. Bioinformatics. 2010;26(19):2460–2461. doi:10.1093/bioinformatics/btq461. 20709691

[66] Caporaso JG, Kuczynski J, Stombaugh J, Bittinger K, Bushman FD, Costello EK, Fierer N, Peña AG, Goodrich JK, Gordon JI, et al. QIIME allows analysis of high-throughput community sequencing data. Nat Methods. 2010;7(5):335–336. doi:10.1038/nmeth.f.303. 20383131 PMC3156573

[67] Price MN, Dehal PS, Arkin AP. FastTree: computing large minimum evolution trees with profiles instead of a distance matrix. Mol Biol Evol. 2009;26(7):1641–1650. doi:10.1093/molbev/msp077. 19377059 PMC2693737

