# Supplementary material for: Bacteroides uniformis combined with fiber amplifies metabolic and immune benefits in obese mice
Source: Gut Microbes. 2021 Jan 26;13(1):1865706. doi: 10.1080/19490976.2020.1865706 (PMC8018257; doi:10.1080/19490976.2020.1865706)
Supplement: Supplemental Material [file KGMI_A_1865706_SM6130.docx]

**
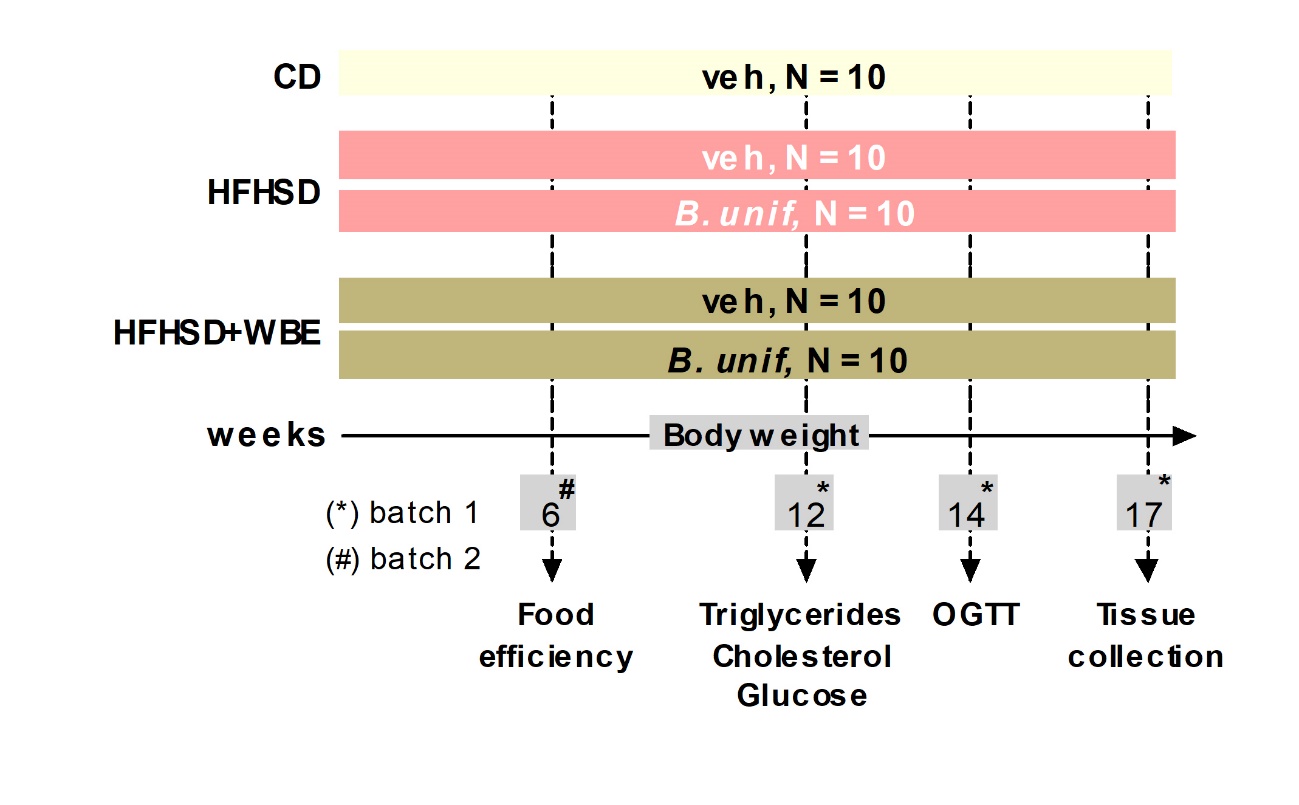
**

**Figure S1. Schematic representation of the experimental procedure**

Mice were fed either control diet (CD: 10% of energy from fat and without sucrose; N = 10), high-fat high-sugar diet (HFHSD: 45% of energy from fat and 35% from sucrose; N = 20) or HFHSD supplemented with 5% of wheat-bran extract (WBE) (HFHSD+WBE; N = 20) for 17 weeks. Both mice fed the HFHSD or HFHSD+WBE were subdivided into two experimental groups. One of these groups received an oral dose of vehicle (veh, N = 10) and the other an oral dose *B. uniformis* CECT 7771 (5x10^7^ CFU per mouse, N = 10) daily. CD-fed mice received only vehicle (N = 10). Body weight was determined weekly. At week 12, glucose, triglycerides and cholesterol were determined in plasma and OGTT was conducted at week 14. Mice were sacrificed at week 17 for blood collection and isolation of tissues (small and large intestine, liver, epididymal WAT, and caecal content). Food efficiency was estimated at week 6 of HFHSD in another batch of animals. CD, control diet; *B. unif*, *Bacteroides uniformis* CECT 7771; HFHSD, high-fat high-sugar diet; OGTT, oral glucose tolerance; WBE, wheat bran extract.


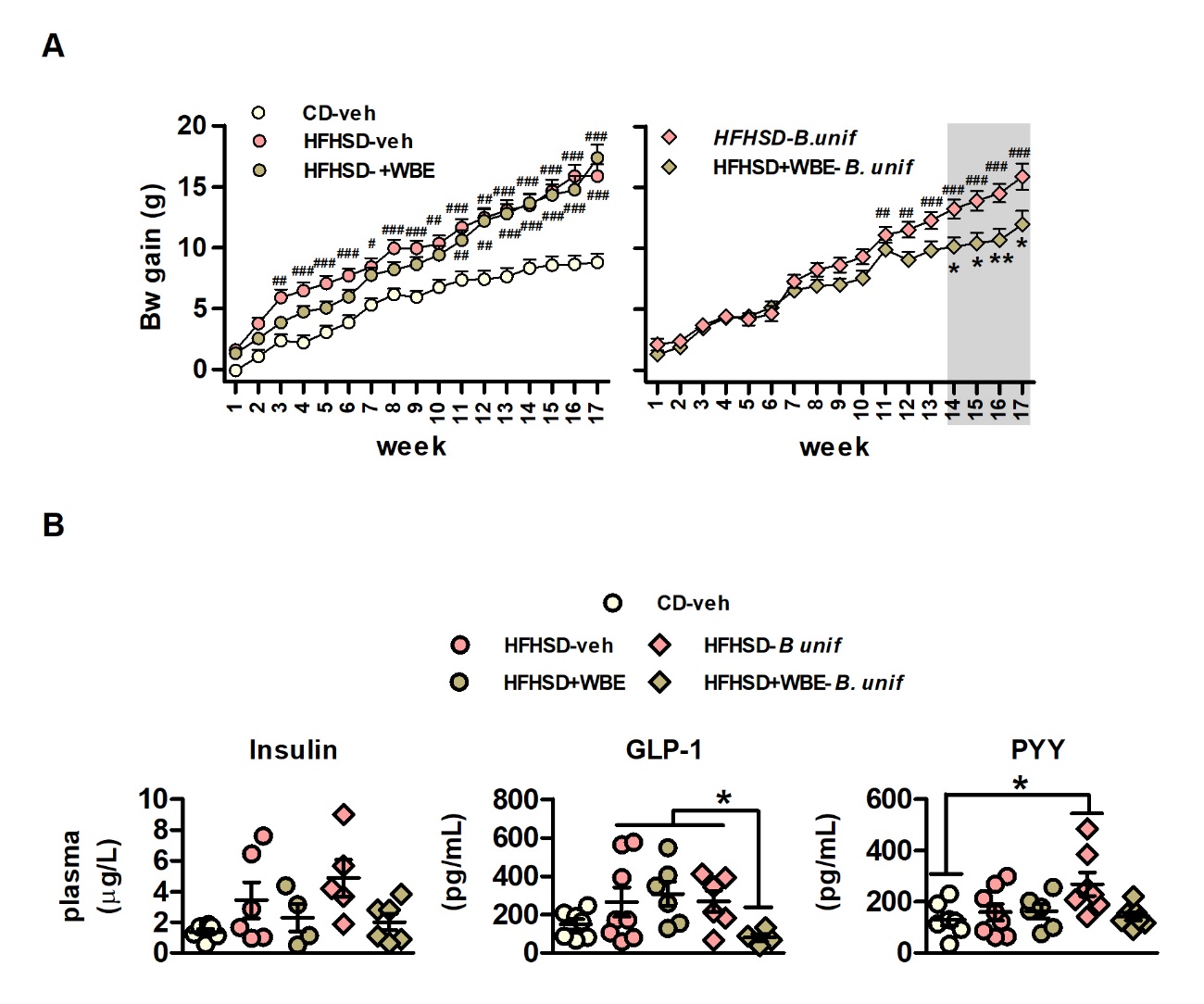


**Figure S2 related to Figure 1. Effects *B. uniformis* CECT 7771 and WBE, separately or combined, on body weight evolution and key hormones controlling energy metabolism.**

Body weight evolution and plasma levels of insulin, GLP-1 and PYY of controls (CD-fed mice receiving vehicle, CD-veh) and HFHSD fed mice receiving vehicle, WBE, *B. uniformis*, or the combination of both: HFHSD-veh, HFHSD+WBE, HFHSD-*B. unif* or HFHSD+WBE-*B. unif*)

1. Body weight evolution from week 1 to 17 (two-way ANOVA HFHSD-fed groups; *B. unif* x WBE interaction P <0.05 at week 14 to 17; *post hoc* P <0.05. N = 10 for all groups
2. Insulin, GLP-1 (Kruskal-Wallis test: HFHSD+WBE-*B. unif* vs HFHSD-veh, HFHSD-*B. unif* or HFHSD+WBE P <0.05) and PYY concentrations in plasma at week 17 (Kruskal-Wallis test: HFHSD+WBE-*B. unif* vs CD-veh P <0.05). Insulin: CD-veh, HFHSD-veh and HFHSD+WBE-*B. unif* N = 6; HFHSD+WBE N = 4 and HFHSD-*B. unif* N = 5. PYY: CD-veh, HFHSD-B. unif and HFHSD+WBE-B. unif N = 7; HFHSD-veh N =8 and HFHSD+WBE N = 6. GLP-1: CD-veh N = 7; HFHSD-veh N = 8; HFHSD+WBE and HFHSD-*B. unif* N = 6 and HFHSD+WBE-*B. unif* N = 4.

Data were represented as the mean ± SEM. ^#^P <0.05, ^##^P <0.01, ^###^P <0.001 vs control group. *P <0.05 indicates differences within HFHSD-fed groups. *B. unif*, *Bacteroides uniformis* CECT 7771, CD, control diet; GLP-1, glucagon-like peptide 1; HFHSD, high-fat high-sugar diet; PYY, peptide YY; veh, vehicle and WBE, wheat bran extract.

**
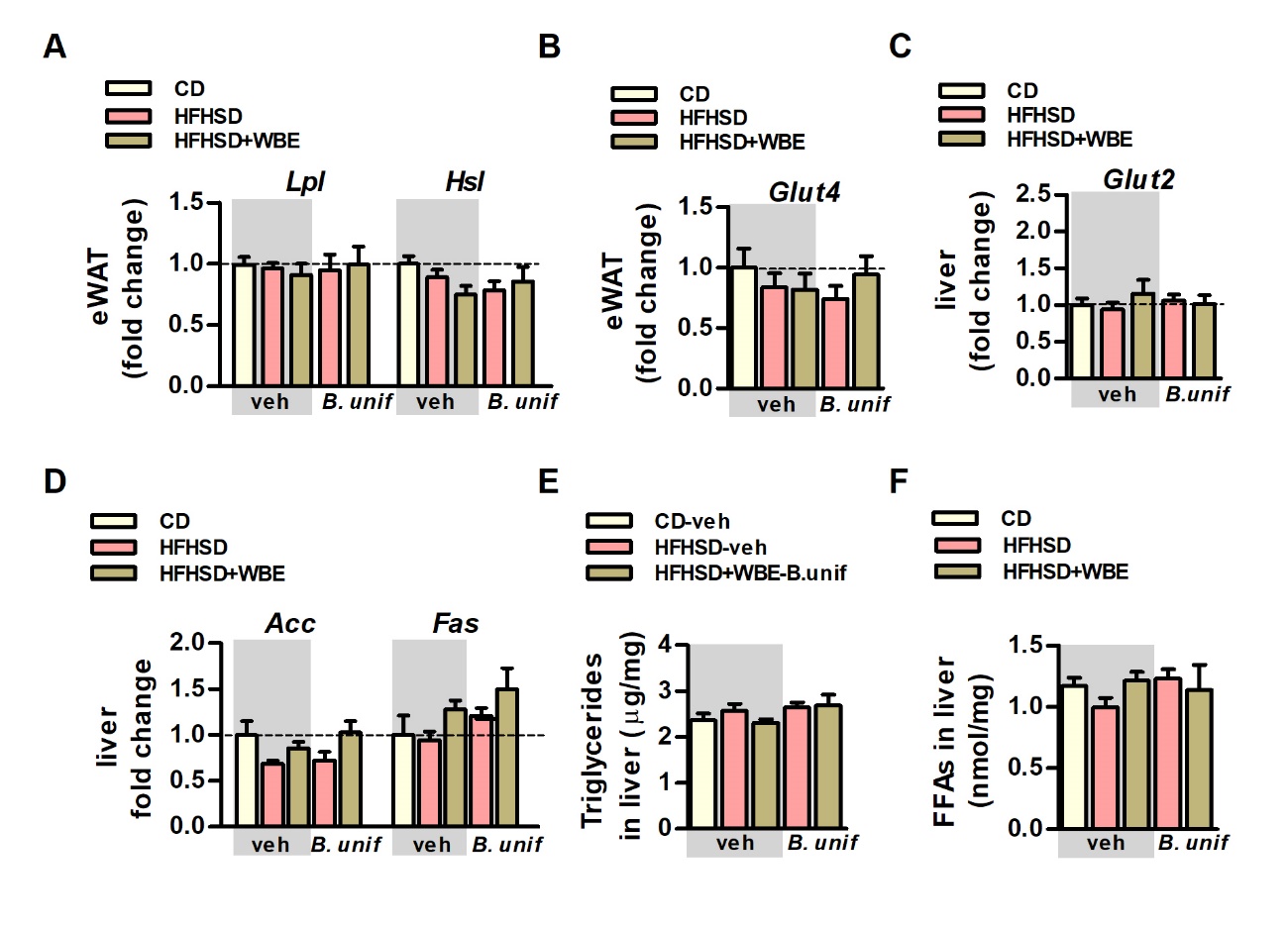
**

**Figure S3. Related to Figure 2. Characterization of markers of lipid metabolism in fat and liver** of controls (CD-fed mice receiving vehicle, CD-veh) and HFHSD-fed mice receiving vehicle, WBE, *B. uniformis* or the combination of both (WBE+*B. unif*): HFHSD-veh, HFHSD+WBE, HFHSD-*B. unif* or HFHSD+WBE-*B. unif*).

1. *Lpl* and *Hsl* gene expression in epididymal WAT at week 17. *LpL*: CD-veh, HFHSD-veh and HFHSD+WBE-*B.unif* N = 7; HFHSD+WBE N = 8 and HFHSD-*B.unif* N = 9. *Hsl*: CD-veh, HFHSD-veh and HFHSD+WBE-*B.unif* N = 7 and HFHSD+WBE and HFHSD-B.unif N = 8
2. Gene expression of *Glut4* in epididymal WAT at week 17. *Glut4*: CD-veh and HFHSD-*B. unif* N = 6 and HFHSD-veh, HFHSD+WBE and HFHSD+WBE-*B. unif* N = 7
3. Gene expression of *Glut2* in liver at week 17. *Gltu2*: CD-veh and HFHSD+WBE N = 6; HFHSD-veh N =6 and HFHSD-*B. unif* and HFHSD+WBE-*B. unif* N = 8.
4. *Acc* and *Fas* gene expression in liver at week 17. CD-veh, HFHSD-veh and HFHSD+WBE-B.unif N = 8; HFHSD+WBE N = 6 and HFHSD+WBE-B.unif N = 7
5. Triglycerides levels in liver at week 17. CD-veh, HFHSD-veh and HFHSD+WBE-*B.unif* N = 10 and HFHSD+WBE and HFHSD-*B.unif* N = 9.
6. Free fatty acids (FFAs) levels in liver at week 17. CD-veh N = 8; HFHSD-veh and HFHSD-*B.unif* N = 9 and HFHSD+WBE and HFHSD-*B.unif* N = 7

Data were represented as the mean ± SEM. Parametric tests (one-way ANOVA to compare controls vs HFHSD-fed mice and two-way ANOVA restricted to HFHSD-fed mice) were conducted to analyse data of panels A and C while Kruskal-Wallis test was used to assessed data of panel B. *Acc*, acetyl-CoA carboxylase; *B. unif*, *B. uniformis* CECT 7771; eWAT, epididymal white adipose tissue; CD, control diet; *Fas*, fatty acid synthase; FFAs, free fatty acids; HFHSD, high-fat high-sugar diet; *Hsl*, hormone sensitive lipase; *Lpl*, lipoprotein lipase; veh, vehicle; WBE, wheat bran extract.

**
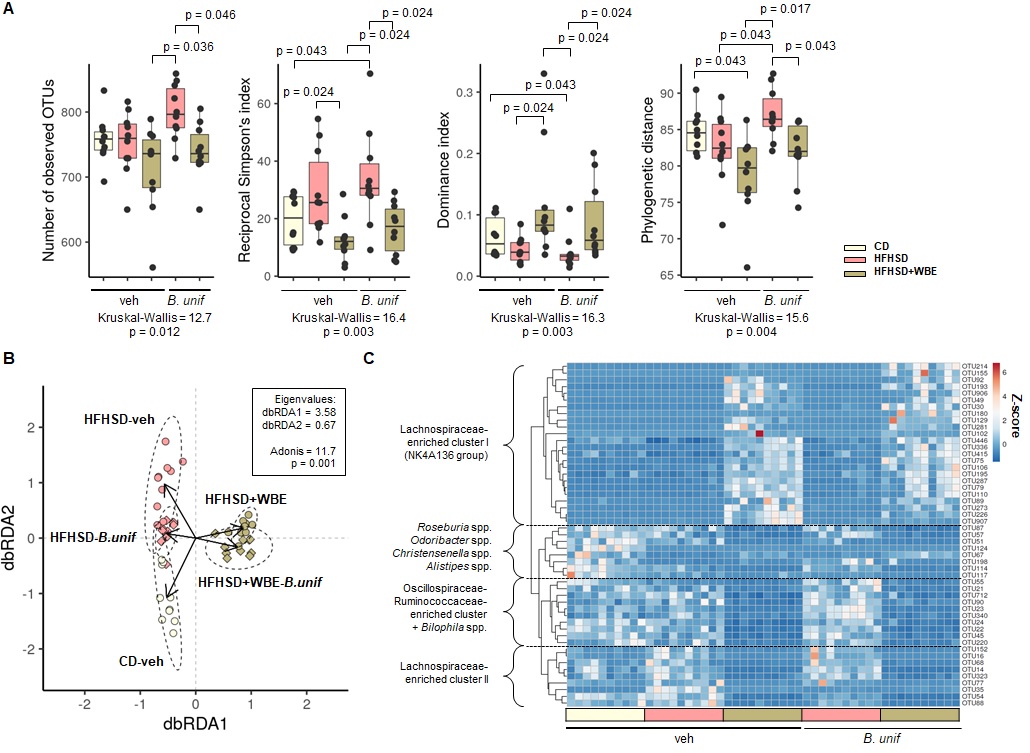
**

**Figure S4**. **Related to Figure 4.** **Effects of *B. uniformis* CECT 7771 and WBE, separately or in combination, on gut microbiota of control and diet-induced obese mice.**

Microbiota analysis of the caecal content at week 17 of controls (CD-fed mice receiving vehicle, CD-veh) and HFHSD fed mice receiving vehicle, WBE, *B. uniformis*, or the combination of both: HFHSD-veh, HFHSD+WBE, HFHSD-*B. unif* or HFHSD+WBE-*B. unif*)

1. The alpha diversity, including study of the observed OTUs, Simpson’s reciprocal index, dominance index, and phylogenetic distance descriptors, was assessed and compared among experimental groups. Alpha diversity data is presented in a boxplot and results of the statistical analysis are shown at the bottom of boxplots, respectively. Pairwise differences between groups are stated at top by showing respective corrected p-values.
2. A beta diversity evaluation of the caecal microbial community structure is provided using distance-based redundancy analysis (dbRDA). The two gradients of dataset dispersion in ordination space with more strength (eigenvalues) of this constrained approach are shown in a scatter-plot. Dashed lines circumscribe the confidence interval (95%) for distribution of respective grouped samples. The eigen values, as well as the result of the adonis test, are depicted in the text box embedded. Arrows’ heads point out the respective centroids of data dispersion. N = 10 for all groups.
3. Scaled read counts for top differentially abundant OTUs (Kruskal-Wallis chi-squared test ≥ 30, corrected P ≤ 0.001) across groups are shown as a heatmap. Clustering of OTUs was carried out by using “correlation” as distance metrics and “complete” as clustering method. The OTUs from major clusters were identified using SINA aligner and taxonomy is presented accordingly. Heat scale is based on Z-scores resulting from rarefied read counts per OTU (raw scaling). N = 10 for all groups. *B. unif*, *Bacteroides uniformis* CECT 7771; CD, control diet; HFHSD, high fat high sugar diet; veh, vehicle; WBE, wheat bran extract. N = 10 for all groups.

**
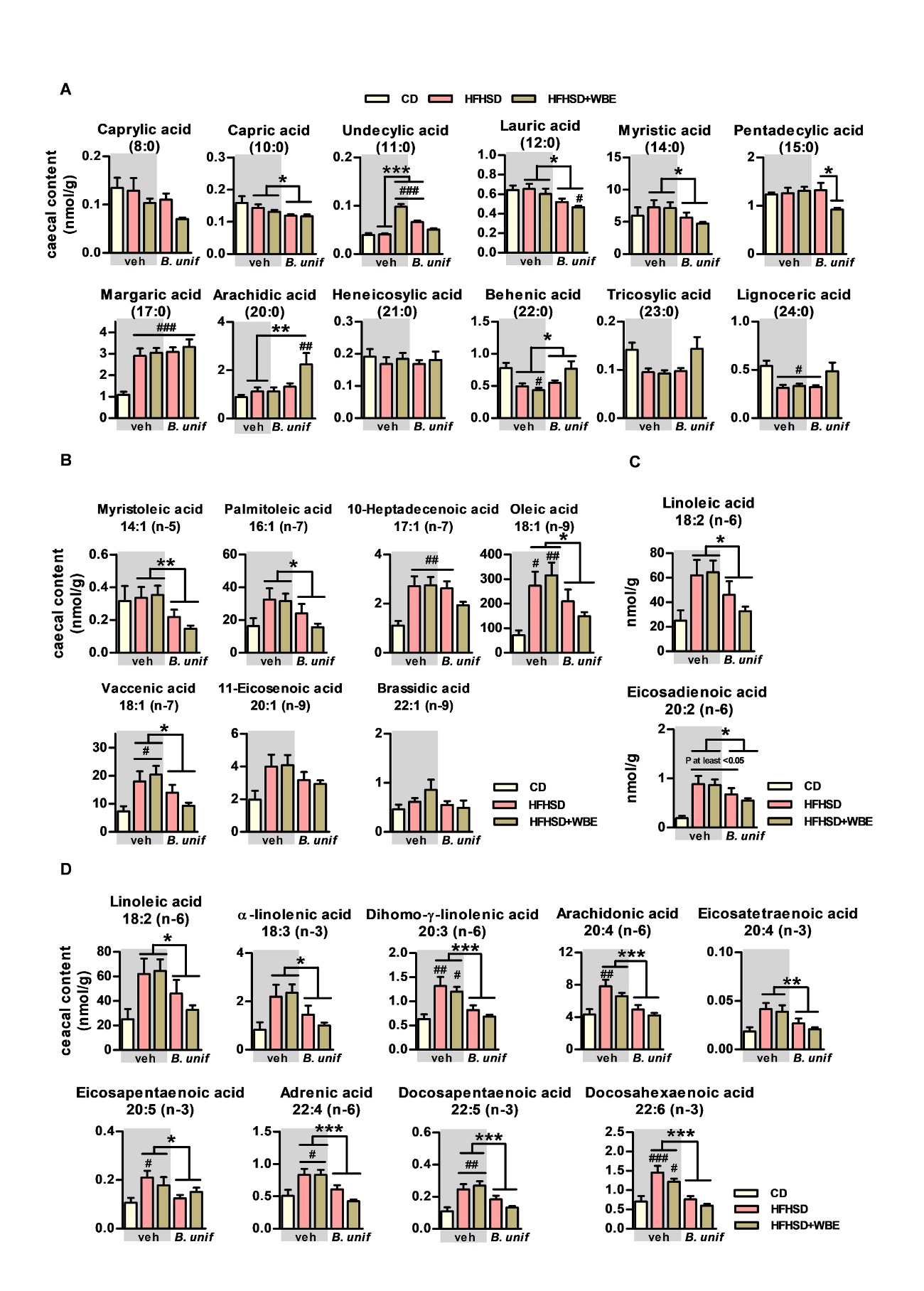
**

**Figure S5. Related to Figure 4. Effects of *B. uniformis* CECT 7771 and WBE, separately or in combination, on caecal long chain fatty acids.**

**A-D.** Saturated FAs, MUFAs, diunsaturated FAs and PUFAs concentrations (nmol/ g of dry weight of caecal content) in controls (CD-fed mice receiving vehicle, CD-veh) and HFHSD-fed mice receiving vehicle, WBE, *B. uniformis* or the combination of both (WBE+*B. unif*): HFHSD-veh, HFHSD+WBE, HFHSD-*B. unif* or HFHSD+WBE-*B. unif*) at week 17. CD-veh N = 8; HFHSD-veh, HFHSD-B-unif, HFHSD+WBE-B. unif N = 10 and HFHSD+WBE N = 9

Data were represented as the mean ± SEM. Parametric tests (one-way ANOVA to compare controls vs HFHSD-fed mice and two-way ANOVA restricted to HFHSD-fed mice) were conducted to analyse all panels. ^#^P <0.05, ^##^P <0.01 and ^###^P <0.01 vs control group. *P <0.05, **P <0.01 and ***P <0.001 indicate differences within HFHSD-fed groups. CD, control diet; *B. unif*, *Bacteroides uniformis* CECT 7771; FAs, fatty acids; HFHSD, high-fat high-sugar diet; MUFAs, monounsaturated FAs; PUFAs, polyunsaturated FAs; veh, vehicle and WBE, wheat bran extract.

**
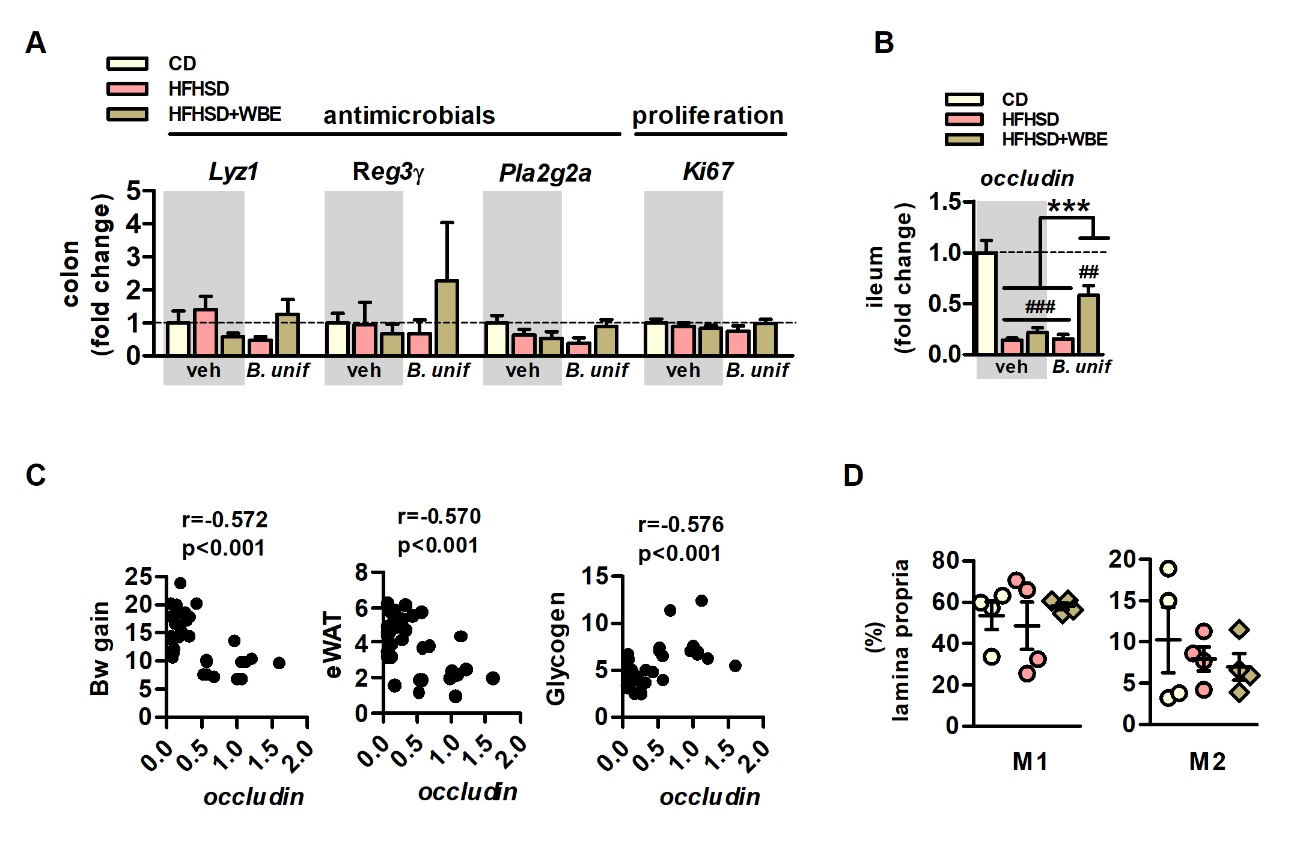
**

**Figure S6. Related to Figure 5. Markers of: defence barrier in colon, intestinal integrity and type 1 and type 2 macrophages in small intestine**

1. Gene expression of *Lyz1*, R*eg3*ɤ, *Pla2g2a* and *Ki67* in colon of controls (CD-fed mice receiving vehicle, CD-veh) and HFHSD-fed mice receiving vehicle, WBE, *B. uniformis* or the combination of both: HFHSD-veh, HFHSD+WBE, HFHSD-*B. unif* or HFHSD+WBE-*B. unif*) at week 17. *Lyz1*: CD-veh, HFHSD+WBE, HFHSD-*B.unif* and HFHSD+WBE-*B.unif* N = 10 and HFHSD-veh N = 9. R*eg3*ɤ: CD-veh and HFHSD-*B.unif* N = 9; HFHSD-veh and HFHSD+WBE-*B.unif* N = 10 and HFHSD+WBE N = 8. *Pla2g2a*: CD-veh, HFHSD-veh and HFHSD+WBE-*B.unif* N = 10; HFHSD+WBE N =8 and HFHSD-*B.unif* N = 9. *Ki67*: CD-veh and HFHSD+WBE-*B.unif* N = 10 and HFHSD-veh, HFHSD+WBE and HFHSD-*B.unif* N = 8.
2. *Occludin* gene expression in ileum of controls (mice fed CD and receiving vehicle, CD-veh N = 7) and HFHSD fed mice receiving or not WBE, *B. uniformis*, or the combination of both: HFHSD-veh, HFHSD+WBE, HFHSD-*B. unif*, or HFHSD+WBE-*B. unif*) (two-way ANOVA in HFHSD-fed groups: *B. unif* x WBE interaction P <0.01; *post-hoc* P <0.001 vs HFHSD-veh, HFHSD+WBE or HFHSD-*B. unif*) at week 17. CD-veh, HFHSD+WBE and HFHSD-*B. unif* N = 7; HFHSD-veh N =8 and HFHSD+WBE-*B. unif* N = 9
3. Bravais-Pearson´s correlation between *occludin* expression in ileum and Bw gain, epididymal fat or hepatic glycogen depots at week 17. N = 38
4. Type 1 and type 2 macrophages in lamina propria (percentage) at week 17 in controls (CD-fed mice receiving vehicle, CD-veh N = 7) and HFHSD fed mice receiving vehicle or the combination of *B. uniformis* and WBE. N = 4

Data were represented as the mean ± SEM. Parametric tests (one-way ANOVA to compare controls vs HFHSD-fed mice and two-way ANOVA restricted to HFHSD-fed mice) were used to analyse *Ki67*, *Tcf4* and occludin and Kruskal-Wallis test to analyse *Lyz1*, R*eg3*ɤ and *Pla2g2a*. *B. unif*, *B. uniformis* CECT 7771; CD, control diet; HFHSD, high-fat high-sugar diet; L*yz1*, lisozyme 1; M1, type 1 macrophages; M2, type 2 macrophages; *Pla2g2a*, phospholipase A2 group IIA; R*eg3ɤ*, regenerating islet-derived protein 3 gamma; veh, vehicle; WBE, wheat bran extract.

**
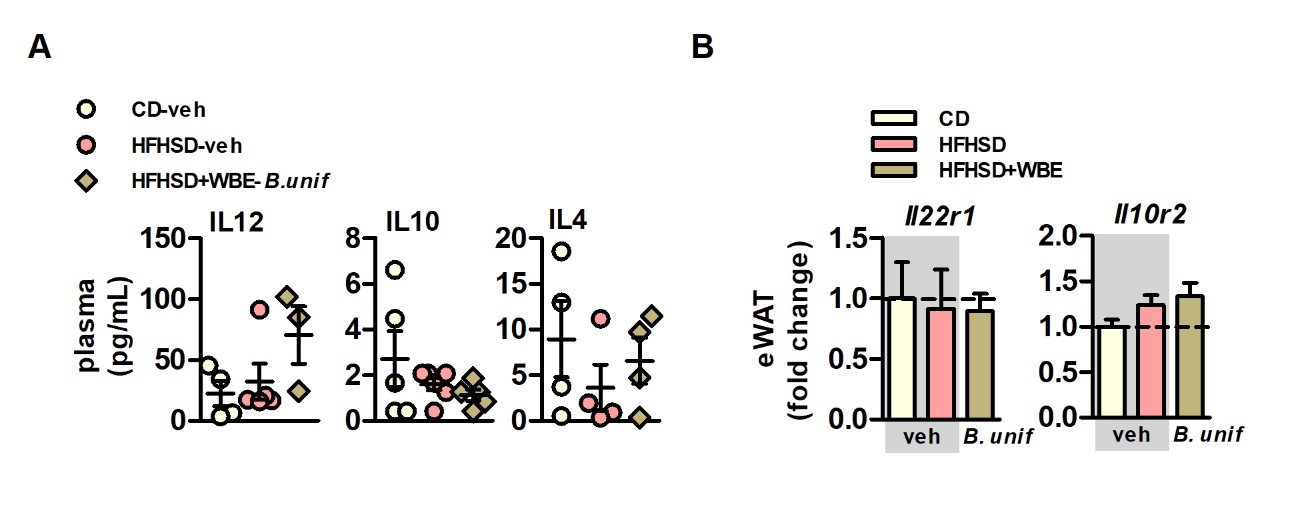
**

**Figure S7.**  **Related to Figure 6. Inflammation markers in plasma and fat**

1. IL12, IL10 and IL4 in plasma of controls (CD-fed mice receiving vehicle, CD-veh) and HFHSD-fed mice receiving vehicle or *B. uniformis* combined with WBE: HFHSD-veh or HFHSD+WBE-*B. unif*) at week 17. IL12: CD-veh N = 4; HFHSD-veh N = 5 and HFHSD+WBE-*B.unif* N = 3. IL10: N = 5 for all groups. IL4: N = 4 for all groups
2. *Il22r1* and *Il10r2* expression in epididymal WAT at week 17. N = 8 for all groups

Data were represented as the mean ± SEM. IL10 and panel B were assessed by one-way ANOVA followed by *post hoc* Tuckey, while Kruskal was used to analyse data of panel *B. unif*, *Bacteroides uniformis* CECT 7771; eWAT, epididymal white adipose tissue; CD, control diet; HFHSD, high-fat high-sugar diet; veh, vehicle; WBE, wheat bran extract.

**Table S1.** Dietary fatty acid composition

| **Fatty acids**  **(FAs)** | **D12450K [%]** | **D12451 [%]** |
| --- | --- | --- |
| **C 12:0** | 0.01 | 0.05 |
| **C 14:0** | 0.04 | 0.29 |
| **C 16:0** | 0.69 | 5.33 |
| **C 18:0** | 0.31 | 2.92 |
| **C 20:0** | 0.02 | 0.07 |
| **C 16:1** | 0.05 | 0.62 |
| **C 18:1** | 1.3 | 9.42 |
| **C 18:2** | 1.43 | 3.46 |
| **C 18:3** | 0.15 | 0.37 |

**Table S2.** Gene names, abbreviations and primer sequences

| Gene name | Abbreviation | Sequence 5´- 3´ | Supplier |
| --- | --- | --- | --- |
| Acetyl-CoA carboxylase | ***Acc*** | Forward: TAATGGGCTGCTTCTGTGACTC  Reverse: CTCAATATCGCCATCAGTCTTG | Isogen Life Science |
| Carbohydrate response element-binding protein alpha | ***ChREBPα*** | Forward: CGACACTCACCCACC  Reverse: TTGTTCAGCCGGATC | Isogen Life Science |
| Carnitine plamitoyltransferase 1a | ***Cpt1a*** | Forward: TTTGAATCGGCTCCTAATGG  Reverse: CCCAAGTATCCACAGGGTCA | Isogen Life Science |
| Fatty acid synthase | ***Fas*** | Forward: GGAGGTGGTGATAGCCGGTAT | Isogen Life Science |
|  |  | Reverse: TGGGTAATCCATAGAGCCCAG |  |
| Glucokinase | ***Gck*** | Forward: ATGTGAGGTCGGCATGATTGT  Reverse: CCTTCCACCAGCTCCACATT | Isogen Life Science |
| Glucose transporter 2 | ***Glut2*** | Forward: TTGTGCTGCTGGATAAATTC  Reverse: AAATTCAGCAACCATGAACC | Sigma-Aldrich |
| Glucose transporter 4 | ***Glut4*** | Forward: CAATGGTTGGGAAGGAAAAG  Reverse: AATGAGTATTCTCATAGGAGGC | Sigma-Aldrich |
| Hormone sensitive lipase | ***Hsl*** | Forward: ATGCCACTCACCTCTGATCC  Reverse: CTGTCCTGTCCTTCCCGTAG | Isogen Life Science |
| Interleukin 22 | ***Il22*** | Forward: GACATAAACAGCAGGTCCAGTT | Isogen Life Science |
|  |  | Reverse: AGAAGGCTGAAGGAGACAGT |  |
| Interleukin 10 receptor type 2 | ***Il10r2*** | Forward: GGACGTCTCTTCCACAGCAC | Isogen Life Science |
|  |  | Reverse: CTGCTTGCTGCCTTCAGACT |  |
| Interleukin 22 receptor type 1 | ***Il22r1*** | Forward: GCTCGCTGCAGCACACTACCAT  Reverse: TGAGTGTGGGGTGGACCAGCAT | Isogen Life Science |
| Ki67 | ***Ki67*** | Forward: CAGACTTGCTCTGGCCTACC  Reverse: GGTTGGCGTTTCTCCTCTTT | Isogen Life Science |
| Lipoprotein lipase | ***Lpl*** | Forward: TGAAAGCCGGAGAGACTCAG  Reverse: AGTGTCAGCCAGACTTCTTCAG | Isogen Life Science |
| Lisozyme 1 | ***Lyz1*** | Forward: GCCAAGGTCTACAATCGTTGTGAGTT  Reverse: CAGTCAGCCAGCTTGACACCACG | Isogen Life Science |
| Occludin | ***occludin*** | Forward: ATGTCCGGCCGATGCTCTC  Reverse: TTTGGCTGCTCTTGGGTCTGTAT | Isogen Life Science |
| Phospholipase A2 group IIA | ***Pla2g2a*** | Forward: AAGGATCCCCCAAGGATGCCAC  Reverse: CAGCCGTTTCTGACAGTTCTGG | Isogen Life Science |
| Regenerating islet-derived protein 3 gamma | ***Reg3ɤ*** | Forward: TTCCTGTCCTCCATGATCAAA  Reverse: CATCCACCTCTGTTGGGTTC | Isogen Life Science |
| Ribosomal protein L19 | ***Rpl19*** | Forward: CCTTGTCTGCCTTCAGCTTGT  Reverse: GAAGGTCAAAGGGAATGTGTTCA | Isogen Life Science |
| Uncoupling protein 1 | ***Ucp-1*** | Forward: ACTGCCACACCTCCAGTCATT  Reverse: CTTTGCCTCACTCAGGATTGG | Isogen Life Science |
